# Cerium oxide nanoparticles: Synthesis methods and applications in wound healing

**DOI:** 10.1016/j.mtbio.2023.100823

**Published:** 2023-10-01

**Authors:** Hamed Nosrati, Morteza Heydari, Mohammad Khodaei

**Affiliations:** a Biosensor Research Center (BRC), Isfahan University of Medical Sciences (IUMS), Isfahan, Iran; b Department of Immune Medicine, University of Regensburg, Regensburg, Germany; c Materials Engineering Group, Golpayegan College of Engineering, Isfahan University of Technology, Golpayegan, Iran

**Keywords:** Cerium oxide nanoparticles, Biosynthesis, Wound healing, Nanocomposite scaffolds, Skin tissue engineering

## Abstract

Wound care and treatment can be critical from a clinical standpoint. While different strategies for the management and treatment of skin wounds have been developed, the limitations inherent in the current approaches necessitate the development of more effective alternative strategies. Advances in tissue engineering have resulted in the development of novel promising approaches for accelerating wound healing. The use of various biomaterials capable of accelerating the regeneration of damaged tissue is critical in tissue engineering. In this regard, cerium oxide nanoparticles (CeO_2_ NPs) have recently received much attention because of their excellent biological properties, such as antibacterial, anti-inflammatory, antioxidant, and angiogenic features. The incorporation of CeO_2_ NPs into various polymer-based scaffolds developed for wound healing applications has led to accelerated wound healing due to the presence of CeO_2_ NPs. This paper discusses the structure and functions of the skin, the wound healing process, different methods for the synthesis of CeO_2_ NPs, the biological properties of CeO_2_ NPs, the role of CeO_2_ NPs in wound healing, the use of scaffolds containing CeO_2_ NPs for wound healing applications, and the potential toxicity of CeO_2_ NPs.

## Highlights

• Cerium oxide nanoparticles (CeO_2_ NPs) could be synthesized through different methods, including physical, chemical and biological methods.
• Biosynthesis of CeO_2_ NPs using plants, microbes and other sources is environmentally friendly and cost-effective.
• CeO_2_ NPs exhibit antioxidant, anti-inflammatory, antibacterial, and angiogenic properties, making them an excellent candidate for wound healing applications.
• CeO_2_ NPs can be incorporated into a variety of scaffolds to accelerate wound healing *in vivo*.
• The incorporation of CeO_2_ NPs into scaffolds could provide controlled or sustained release systems, thereby avoiding the nanoparticles' potential dose-dependent toxicity.

## Graphical abstract

**Figure:**
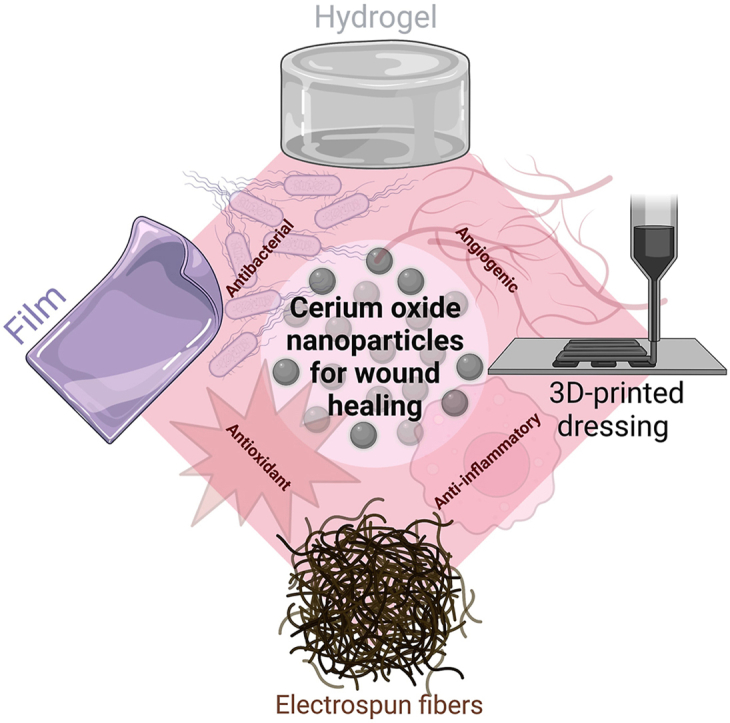

## Statement of significance

CeO_2_ NPs have emerged as a promising candidate for biomedical applications due to their unique features, including antioxidant, anti-inflammatory, angiogenic, and antibacterial properties. While the management of chronic wounds is considered a clinically critical issue, the use of CeO_2_ NPs for wound healing applications has recently piqued the attention of researchers. The *in vivo* results have shown the ability of CeO_2_ NPs to accelerate wound healing by promoting angiogenesis, reducing inflammation, and preventing infections. Additionally, CeO_2_ NPs-incorporated scaffolds have been demonstrated to effectively support the multifactorial nature of the wound healing process. In this review paper, we focus on CeO_2_ NPs synthesis methods, the biological properties of these nanoparticles that make them suitable for wound healing applications, and current practical strategies to take advantage of such properties for wound healing purposes.

## 1 Introduction

The skin is the body's largest and heaviest organ, serving as a protective barrier against external agents. It protects the tissues beneath it while also detecting external stimuli, regulating temperature, preventing dehydration by retaining moisture, and aiding in vitamin D_3_ synthesis. The skin's ability to perform these functions depends on its structural integrity, which can be compromised by various factors including trauma, disease, and surgical incisions [1,2]. A skin wound can be defined as any type of injury to the skin that disrupts the normal structure and function of the skin [3]. Skin wounds can range from minor cuts and scrapes to more severe injuries such as puncture wounds, lacerations, and burns. When the skin is severely injured, it can put human health and even life at risk. Therefore, regeneration is critical in restoring body homeostasis [2,4].

Cutaneous wounds can be divided into two categories: acute and chronic. Acute wounds are commonly caused by burns, mechanical trauma, or surgical incisions; they tend to heal more quickly than chronic wounds. Chronic wounds, however, can take more than three months to heal and are often accompanied by inflammatory responses and significant scarring. Diabetic foot ulcers, venous ulcers, pressure ulcers, arterial ulcers, wounds caused by aging, and chronic wounds resulting from the poor healing of acute wounds are some examples of chronic wounds [5]. Chronic wounds are a significant clinical concern, as they account for approximately 35% of wounds and place a financial burden on both patients and the medical system [5,6].

In recent years, various methods have been developed for the treatment of chronic wounds. Among these methods, the use of skin autografts is considered the preferred approach and is regarded as the gold standard. This technique involves harvesting the epidermis and parts of the underlying dermis from areas of the patient's healthy skin; then the harvested tissue is transplanted onto the wound site [7,8]. However, this method faces some limitations, as it is not feasible for wounds that exceed 60% of the patient's total body surface area. Insufficient and delayed dermis regeneration at the site where the tissue is harvested could result in severe scarring. Hair regeneration problems, pigmentation disturbances, and pain are other complications associated with the donor site [9,10]. While the use of skin autografts is currently considered an effective treatment for chronic wounds, it is not always the best option for all patients. Given the limitations of the current approaches, it is, therefore, necessary to explore alternative strategies and conduct research to address clinical concerns.

Tissue engineering and regenerative medicine are rapidly growing fields that offer promising approaches as effective treatments for hard-to-heal skin wounds [11]. Wound healing is a complex process that involves the coordinated activity of various cells, proteins, and signaling molecules [12]. While traditional wound healing methods have been successful in treating many types of wounds, tissue engineering and regenerative medicine offer promising alternative approaches for more severe or chronic wounds. These approaches involve the use of biomaterials, stem cells, growth factors, and other biological components to accelerate the healing process [13,14]. Along with advances in tissue engineering and regenerative medicine, nanotechnology has emerged as a promising field in the development of innovative approaches for wound healing. The unique properties of nanomaterials, such as their nanoscale size, high surface area to volume ratio, and tunable surface chemistry, make them promising candidates for applications in wound healing. Nanotechnology-based approaches have shown great potential in enhancing wound healing outcomes by promoting cell proliferation and migration, reducing inflammation, inducing angiogenesis, and demonstrating broad-spectrum antimicrobial activity [[15], [16], [17]].

**Fig. 1:**
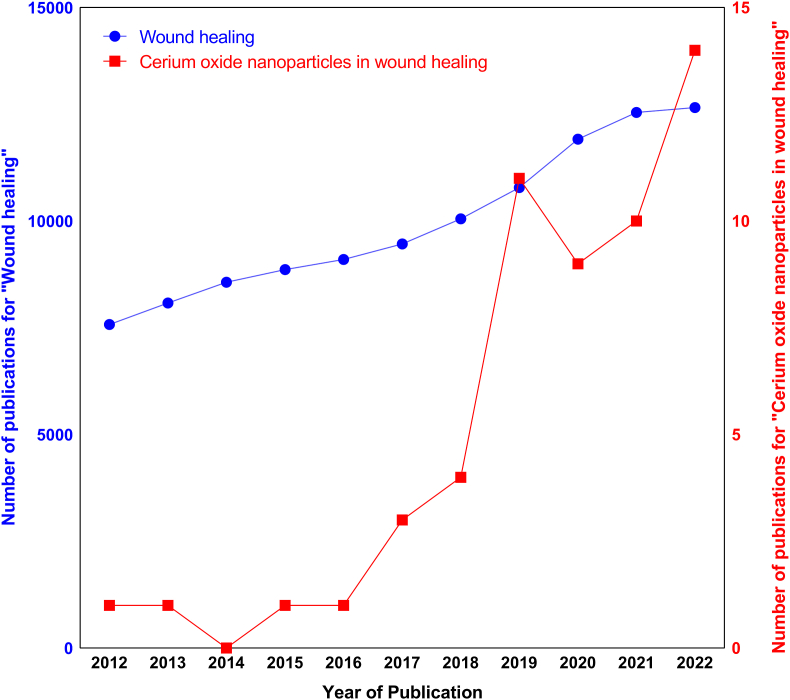
The number of publications on “Wound healing” and “Cerium oxide nanoparticles in wound healing” per year from 2012 to 2022, according to the PubMed database.

Over the past few years, considerable attention has been given to the potential biomedical applications of cerium oxide nanoparticles (CeO_2_ NPs). Accordingly, a wide range of studies have focused on the development of novel CeO_2_ NPs synthesis methods, among which biosynthesis of CeO_2_ NPs using plants, microbes, and food-based products has piqued the attention of researchers [18]. Despite this, research on the use of CeO_2_ NPs for wound healing applications is limited, although a growing trend could be inferred according to the PubMed database (Fig. 1). CeO_2_ NPs have been shown to possess excellent biological properties, including antibacterial, anti-inflammatory, anti-oxidant, and angiogenic properties, which are all desirable for wound healing applications [19,20]. Moreover, the incorporation of CeO_2_ NPs into engineered scaffolds could effectively enhance the efficacy of treatment by providing sustained release systems. This would ensure the exposure of healing tissue to CeO_2_ NPs released during the wound healing process while avoiding the potential toxicity of the high concentrations of these nanoparticles [21].

The following sections will cover the structure and functions of the skin, the wound healing process, current methods for the synthesis of CeO_2_ NPs, the biological properties of CeO_2_ NPs, the role of CeO_2_ NPs in wound healing, the use of scaffolds containing CeO_2_ NPs for wound healing applications, and the potential toxicity of CeO_2_ NPs.

## 2 The skin structure and functions

The skin is the largest and heaviest organ of the human body. It serves very important functions, such as regulating body temperature, detecting external stimuli, and synthesizing vitamin D_3_ (cholecalciferol) from 7-dehydrocholesterol [22]. One of the most important roles of the skin is to act as a barrier, physically protecting the body from the outside world. It prevents water and electrolyte loss, reduces penetration of chemicals, absorbs radiation from the sun, and protects the body from the invasion of pathogenic microbes. The skin also acts as an active immune organ. The production of antimicrobial peptides provides a chemical barrier against pathogenic microbes [23,24].

The skin is a dynamic organ with a complex and integrated structure consisting of two main layers: the epidermis and the dermis. The epidermis, which is the outermost layer of the skin, has a high potential for regeneration. The four main layers of the epidermis from the innermost layer to the outermost are stratum basale (also known as stratum germinativum), stratum spinosum, stratum granulosum, and stratum corneum (also known as cornified layer or horny layer). A fifth layer, called stratum lucidum, could be observed in the thick skin of the soles of the feet and palms of the hands between the stratum corneum and the stratum granulosum. It reduces the friction between these two layers [25,26].

The stratum basale consists of cuboidal to columnar cells that continuously undergo cell division to produce keratinocytes. Keratinocytes are the main epidermal cells, constituting about 90% of epidermal cells. Melanocytes, Langerhans cells, and Merkel cells are other cell types found in the epidermis [27]. Keratinocytes in the stratum basale are connected to each other by desmosomes and to the basement membrane by hemidesmosomes. The stratum spinosum is typically 5 to 10 cell layers thick. Desmosomes are responsible for the adhesion of keratinocytes to each other in this layer. Irregular-shaped keratinocytes that contain highly basophilic keratohyalin granules are found in the stratum granulosum. As keratinocytes mature and migrate to the horny layer, they undergo organelles and nucleus loss. The keratinocytes in the horny layer, also known as corneocytes, are completely keratinized. The desmosomes also begin to disappear or lose their functionality in this layer [28,29].

The dermis is the inner layer of the skin, found deep to the epidermis and superficial to the subcutaneous tissue (hypodermis). It is responsible for approximately 90% of the skin's weight. Blood vessels, sensory neurons, sebaceous glands, hair follicles, and sweat glands are typically found in this layer [22]. There are many cell types in this layer, such as endothelial cells, mast cells, macrophages, smooth muscle cells, adipocytes, and fibroblasts. Fibroblasts are the dominant cells of the dermis. They are responsible for the synthesis of collagen, elastic and reticular fibers, and ECM components. The excellent mechanical properties and elasticity of the skin are primarily attributed to the presence of ECM components, such as collagen and elastin, in the dermis [26,30]. Fibroblasts also play an important role in wound healing [2,31].

The hypodermis, also known as subcutaneous tissue or superficial fascia, lies under the dermis; it consists of fat and loose connective tissues. Adipocytes, fibroblasts, and macrophages are the main cells of the subcutaneous tissue. Larger blood vessels and nerves are located in this layer. The hypodermis stores energy while also acting as a heat insulator and shock absorber. Subcutaneous tissue is also regarded as an endocrine organ in which the aromatase enzyme is involved in the conversion of androstenedione to estrone. In addition, the adipose cells produce leptin, a hormone that mediates the long-term regulation of energy balance [30,32].

## 3 Wounds and the wound healing process

A skin wound is defined as a break in the integrity of the skin that affects its functions. These wounds can occur for various reasons, such as incisions, chemical agents, burns, pathological conditions, etc. They can also be accompanied by disruption of the structure and function of deeper tissues [33]. Skin wounds are generally classified into two types: acute and chronic [2]. Acute wounds have a short healing time and show signs of healing in less than three months. Trauma, surgical incisions, and burns are common causes of acute wounds. The healing of these wounds progresses through the normal phases of the wound healing process, as will be discussed later. Chronic wounds, on the other hand, take more than three months to heal and often exhibit severe inflammation and scarring. Diabetic foot ulcers, pressure ulcers, venous ulcers, and arterial insufficiency ulcers are examples of such wounds. Chronic wounds can also result from the poor healing of acute wounds [5,34]. Approximately 35% of wounds are classified as chronic [35]. Chronic wounds are considered clinically important due to their increasing incidence and greater recognition of their associated morbidity and socioeconomic burden [5,36].

Because of the critical functions of the skin, its integrity must be restored quickly and efficiently. Wound healing is a dynamic and highly regulated process that regenerates the injured skin through four overlapping stages: hemostasis, inflammation, proliferation, and remodeling [37].

a) Hemostasis: The first phase of the wound healing process is hemostasis, which is the immediate reaction of the body to the injury. This phase lasts a few minutes, and its primary aim is to stop the bleeding. Platelets have a critical role in this phase. Activation of platelets promotes the formation of a fibrin clot. They also produce cytokines and growth factors, which aid in the regulation of the healing cascade [26]. The fibrin clot is a temporary structure that must be formed in order to stop bleeding [38]. It also acts as a scaffold for the infiltration of cells involved in the wound healing process [39].
b) Inflammation: The inflammatory phase, the main aim of which is to prevent infection, occurs simultaneously with the hemostasis phase and can last up to 48 h after injury [40]. Infiltration and migration of neutrophils (involved in removing bacteria, foreign particles, and cell debris), lymphocytes, and monocytes (which differentiate into macrophages) occur in this phase. These cells protect the wound site against bacterial infections by producing reactive oxygen species (ROS) and proteinases. Growth factors and cytokines secreted by inflammatory cells initiate the proliferative phase of wound healing [39].
c) Proliferation: The proliferative phase occurs approximately 2–10 days after the damage. Cell proliferation, angiogenesis, granulation tissue formation, collagen deposition, re-epithelialization, and wound contraction all occur during this complex phase [40]. Angiogenesis refers to the formation of new capillaries from pre-existing vessels to form a complex network of blood vessels [15]. Vascular endothelial growth factor (VEGF) is the most important growth factor involved in the angiogenesis process. VEGF is released in response to hypoxia, and its activity in combination with a number of cytokines induces endothelial cells to proliferate, migrate, and form tubes [40,41]. Matrix metalloproteases (MMPs), a family of calcium-dependent zinc-containing endopeptidases, promote angiogenesis through ECM remodeling and releasing ECM-bound proangiogenic factors [42]. As the angiogenesis process proceeds, capillary sprouts invade the fibrin clot to form a rich vascular network of capillaries. The vascular network, along with macrophages and fibroblasts, aids in the formation of granulation tissue [26]. Fibroblasts gradually secrete ECM components such as type III collagen, glycosaminoglycans (GAGs), and proteoglycans. Collagen deposition and alignment are also crucial for skin integrity recovery. Once sufficient ECM has been secreted, some fibroblasts are stimulated by macrophages to differentiate into a myofibroblast phenotype [43]. Myofibroblasts are contractile cells assisting in wound contraction. These cells are also involved in collagen deposition and angiogenesis during wound healing [44,45]. Granulation tissue is finally replaced by normal tissue during the next phase of healing.
d) Remodeling: The final phase of the wound healing process is remodeling, which begins 2–3 weeks after injury and can last up to 2 years, resulting in scar tissue maturation and the development of normal epithelium. In this phase, many myofibroblasts, macrophages, and endothelial cells undergo apoptosis [22,46]. Type III collagen is replaced by type I collagen, increasing tissue tensile strength. Despite this, the repaired tissue will never regain the tensile strength of the healthy tissue before the injury [26,47].

**Fig. 2:**
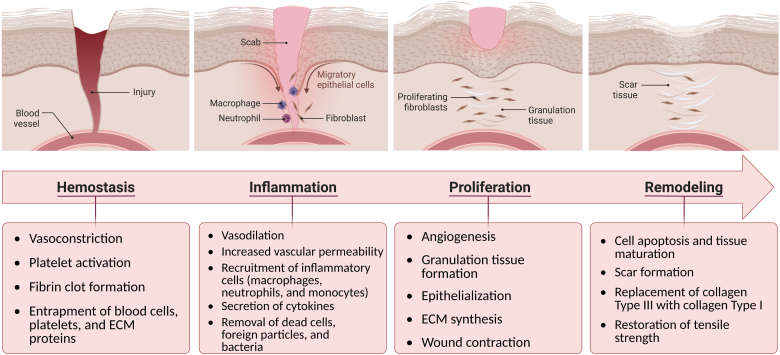
The wound healing process and the key events during each healing phase.

Fig. 2 illustrates the four stages of wound healing, accompanied by the key events taking place during each stage.

## 4 Physical and chemical methods for CeO_2_ NPs synthesis

Various physical and chemical methods can be used for the synthesis of nanoparticles. In this section, we will review some of the most important and widely used methods for the synthesis of CeO_2_ NPs.

### 4.1 Ball milling method

Ball milling is a simple mechanical technique commonly used to fracture the particles and to reduce their size. This technique is typically performed using a hollow cylindrical shell partially filled with balls that can rotate around its axis. The balls are made of materials such as stainless steel, rubber, or ceramics. The process of ball milling relies on the energy released from the attrition between the balls and the powder [48,49]. This technique is fast, eco-friendly, reproducible, and cost-effective. It is also an easy technique to perform and has been used for the synthesis of CeO_2_ in the nanoscale [18]. Yadav and Srivastava reported the synthesis of nanoscale CeO_2_ using high-energy ball milling. The prepared particles were nearly spherical in shape, with an average particle size of 10 nm [50]. Hadi et al. synthesized CeO_2_ NPs through a mechanochemical process. For this purpose, cerium(III) carbonate hydrate (Ce_2_(CO_3_)_3_.xH_2_O) and sodium hydroxide (NaOH) were mixed at a weight ratio of 4:1. The mixtures were then milled for 24 h at room temperature. The powders were washed with ethanol and distilled water, dried for 24 h, and then calcined for 5 h at 100, 350, 500, 650, or 800 °C. The results showed that increasing the annealing temperature from 350 to 800 °C led to an increase in the crystallite size of the nanoparticles, from 4.5 to 20 nm. Higher temperatures resulted in better crystalline CeO_2_ formation; however, the crystallite size was increased due to grain growth [51]. Despite the aforementioned advantages of the ball milling technique, there are also some drawbacks and limitations, the most significant of which are the possibility of contamination, the tendency of nanoparticles to agglomerate, the formation of irregular-shaped nanoparticles, and the long time required for milling and cleaning [18,48].

### 4.2 Precipitation and co-precipitation methods

Precipitation method is the most commonly used method for the synthesis of CeO_2_ NPs. In this method, cerium nitrate hexahydrate is usually used as the precursor for CeO_2_ NPs synthesis in the presence of bases, such as ammonia solution, ammonium carbonate ((NH_4_)_2_CO_3_), sodium hydroxide (NaOH), and potassium carbonate (K_2_CO_3_) [[52], [53], [54]]. Tsai used ammonium cerium nitrate and urea as precursors for the homogeneous precipitation of CeO_2_ NPs with a primary particle size of about 8 nm [55]. Renuka synthesized CeO_2_ NPs by the homogeneous precipitation of a cerium nitrate hexahydrate solution (Ce(NO_3_)_3_·6H_2_O) with dilute ammonia solution. Cerium nitrate hexahydrate was dissolved in ddH_2_O and precipitated by the dropwise addition of the obtained solution to the ammonia solution. Water-soluble species were then removed by deionized water washing. Ethanol was then used to remove water and disrupt hydrogen bonded networks between water and the precursor. The obtained precipitate was dried at 383 K for 12 h, crushed with an agate mortar, and then calcined at 500 °C for 3 h. According to SEM and TEM analyses, the synthesized nanoparticles were spherical in shape with an average particle size of 5.1 nm, which is desirable for many applications [56]. In a study conducted by Farahmandjou et al. CeO_2_ NPs were synthesized by a simple co-precipitation method using cerium(III) nitrate hexahydrate and potassium carbonate as the precursors. Aqueous solutions of cerium nitrate and potassium carbonate were prepared and used to obtain cerium(III) carbonate precipitate. Also, pH was 6 during the precipitation. The resulting product was dried at 65 °C for 2 h, aged at 220 °C for 2.5 h, and then calcined at 600 °C for 3 h to produce CeO_2_ NPs with an average particle size of about 20 nm, as estimated by the XRD technique [57]. The co-precipitation method typically produces nanoparticles with a smaller size distribution and higher purity. This is because the simultaneous mixing of the precursor and the precipitating agent allows for more homogeneous nucleation and growth of the particles. However, the co-precipitation method is generally more complex and time-consuming than the precipitation method [[58], [59], [60]].

### 4.3 Hydrothermal method

The hydrothermal method is also one of the most common methods for the synthesis of CeO_2_ NPs. The hydrothermal method is a technique used for the synthesis of nanoparticles by utilizing high-pressure, high-temperature conditions in a sealed container. Water is used as a solvent in this method, and the chemical reaction occurs within an autoclave [52,61]. In 1993, Zhou and Rahman synthesized CeO_2_ NPs by a hydrothermal method using cerium(III) nitrate hexahydrate as the starting material. Cerium nitrate was dissolved in distilled water, and an ammonium hydroxide solution was added through vigorous stirring to form a precipitate; it was then filtered and washed with distilled water, resulting in the formation of a low-viscosity gel. The gel was placed in a Teflon tube, which was then sealed and placed in an autoclave. A pressure of about 10 MPa and a temperature of about 300 °C were then applied for 4 h. The obtained CeO_2_ powder was washed with distilled water, dried at room temperature, crushed in a mortar and pestle, and then dried again for 2 h at 200 °C. According to TEM micrographs, the average particle size of the synthesized CeO_2_ NPs was 14 ± 3 nm [62]. Renu et al. used cerium chloride as the precursor for hydrothermal synthesis of CeO_2_ NPs. The nanoparticles, which had a size range of 100–200 nm, were found to be nontoxic to mouse fibroblast L929 cells [63]. Masui et al. applied a modified hydrothermal process to produce ultrafine and well-dispersed CeO_2_ NPs from cerium chloride hexahydrate, citric acid, and ammonia solution as the precursors. Citric acid was used as a protective agent to inhibit particle growth [64].

### 4.4 Sol-gel method

The sol-gel method is another widely used technique for the synthesis of CeO_2_ NPs. It involves the formation of a sol, which is a colloidal suspension of particles in a liquid, followed by the gelation of the sol to form a gel. The obtained gel will then be transformed to solid by calcination [65,66]. The sol-gel method for the synthesis of CeO_2_ NPs typically involves the following steps [[67], [68], [69]]:

a) Preparation of the sol: The cerium precursor is dissolved in a suitable solvent to form a homogeneous solution. This solution is then mixed with a stabilizing agent, such as a surfactant or a polymer, to prevent the particles from agglomerating.
b) Hydrolysis and condensation: The sol is then subjected to hydrolysis and condensation reactions, which can result in the formation of CeO_2_ NPs. Hydrolysis involves the reaction of the cerium precursor with water, while condensation involves the formation of chemical bonds between cerium-oxygen atoms.
c) Aging: The resulting gel is allowed to age for a period of time, typically several hours to several days. During aging, the gel becomes denser, and the particles become more uniform in size and shape.
d) Drying: The gel is then dried to remove the solvent and any remaining water. This can be done by evaporating the solvent at low temperatures or using supercritical drying techniques.
e) Calcination: The dried gel is then heated at a high temperature, typically above 500 °C, to dehydrate the dried gel. This process also removes any remaining organic materials and stabilizers.

In a recent study done by Ioannou et al. CeO_2_ NPs were synthesized using the sol-gel method. The researchers used gelatin as the stabilizing agent and cerium nitrate hexahydrate (Ce (NO_3_)_3_•6H_2_O) as the precursor. The study aimed to investigate the effect of changing the ratio of cerium precursor to gelatin on the synthesized CeO_2_ NPs properties. According to the findings, as the amount of cerium precursor was increased and that of gelatin was decreased, the particles grew larger, thus highlighting the importance of using the appropriate amount of stabilizer during the sol-gel synthesis of CeO_2_ NPs [70].

Overall, the sol-gel method is a versatile technique that can be used to synthesize CeO_2_ NPs with a wide range of sizes and shapes. It also allows for precise control of the composition and purity of the nanoparticles. However, the process can be time-consuming and requires careful control of reaction conditions [71,72].

### 4.5 Solution combustion method

The solution combustion method is a simple and effective method for the synthesis of CeO_2_ NPs. It involves the combustion of a solution containing cerium nitrate or cerium chloride and a fuel such as urea or glycine. The combustion reaction is exothermic, and the heat generated is sufficient to drive the formation of CeO_2_ NPs. The reaction occurs in a matter of seconds, and the resulting product is a fine powder of CeO_2_ NPs [73,74]. Zarezadeh Mehrizi et al. synthesized CeO_2_ NPs by the combustion of aqueous solutions containing the corresponding cerium nitrate, ammonium nitrate, and glycine redox mixtures. The obtained particles were spherical in shape and had a size range of 20–30 nm. The *in vitro* cytotoxicity tests on L929 cells demonstrated that there was no toxic effect at any concentration, including the highest concentration of 1000 μg/mL. These results, therefore, indicated that the synthesized nanoparticles were biocompatible and had the potential for use in a variety of biomedical applications [75]. In a study conducted by Ghahramani et al. CeO_2_ NPs were synthesized by the solution combustion method. Cerium nitrate hexahydrate was used as the precursor, while urea, glycine, glucose, and citric acid were used as fuels. According to the findings, the type of fuel used had an impact on the microstructure and crystallinity of CeO_2_ powders due to variations in combustion heat. Glycine fuel generated the highest volume of exhaust gas and exhibited superior combustion, leading to the formation of well-crystallized nanoparticles. Additionally, the results of the corrosion tests indicated that the samples produced with urea fuel were capable of releasing a higher amount of Ce^+4^ ions, hence providing better corrosion protection [74].

Overall, the solution combustion method is simple and cost-effective for the synthesis of CeO_2_ NPs. It also has a short reaction time and requires relatively low temperatures, making it a fast method. However, this method requires careful control of experimental conditions in order to avoid unwanted side reactions and obtain nanoparticles with the desired properties [73,76].

### 4.6 Sonochemical method

The synthesis of nanoparticles using sonochemistry has been of much interest due to the environmentally friendly nature of this approach. This process involves utilizing high-frequency sound waves to generate cavitation bubbles within a liquid medium. This, in turn, leads to the production of high local temperature and pressure, which can trigger a chemical reaction that ultimately yields nanoparticles [77,78]. The sonochemical method can be used for the synthesis of CeO_2_ NPs. Kusuma et al. reported the sonochemical synthesis of CeO_2_ NPs using cerium nitrate hexahydrate and sodium hydroxide as precursors. For this purpose, the precursor solution was prepared by mixing a 1.0 M solution of cerium nitrate hexahydrate with deionized water and slowly adding a 0.5 M solution of sodium hydroxide dropwise while stirring continuously. The resulting mixture was further agitated with the dropwise addition of 0.5 M sodium hydroxide. The solution was then sonicated for 1h at 40 °C. The resulting precipitate was filtered using Whatman filter paper and washed with distilled water and ethanol to remove excess sodium hydroxide. The precipitate was then dried for 1h at 100 °C in a hot air oven before being calcined for 2 hours at 1000–1100 °C to obtain CeO_2_ NPs. The synthesized CeO_2_ NPs were characterized and found to have a crystallite size ranging from 35 to 38 nm. The nanoparticles exhibited high efficiency in the photocatalytic degradation of the industrial dye methylene blue under UV light irradiation; they also demonstrated high sensitivity in the detection of paracetamol [79].

In a study by Yin et al. CeO_2_ NPs were synthesized sonochemically using cerium nitrate and azodicarbonamide as the starting materials. Ethylenediamine, tetraethylammonium hydroxide, tetrabutylammonium hydroxide, or tetramethylammonium hydroxide were used as the additives. The presence of additives was found to significantly affect the particle size and particle size distribution. Smaller and more uniform particles were obtained with the use of additives, as compared to highly agglomerated particles obtained without additives. The study also found that the use of tetramethylammonium hydroxide as an additive and a molar ratio of 1:1:1 of cerium nitrate/azodicarbonamide/tetramethylammonium hydroxide resulted in the production of monodispersed CeO_2_ NPs with an average particle size of approximately 3.3 nm [80].

### 4.7 Microemulsion method

Microemulsions are thermodynamically stable, optically transparent, and isotropic dispersions of two immiscible liquids, one being polar (e.g., water) and the other apolar (e.g., an organic oil), stabilized by a surfactant or a mixture of surfactants. Microemulsions are formed by the spontaneous self-assembly of the surfactant molecules at the oil-water interface, which can reduce the interfacial tension and promote the formation of small droplets of the dispersed phase. Because of their small size and high surface area, microemulsions have unique physicochemical properties that make them useful for a wide range of applications [[81], [82], [83], [84]]. A variety of nanoparticles can be synthesized using the microemulsion method. This method enables the control of particle properties such as size, geometry, morphology, homogeneity, and surface area [85].

In a study done by Masui et al. CeO_2_ NPs were synthesized by the microemulsion method. In this study, a microemulsion containing cerium salt was prepared by mixing polyoxyethylene(10) octylphenyl ether with n-hexyl alcohol and adding it to cyclohexane. Then, a cerium nitrate aqueous solution was added to the resulting mixture. Another microemulsion containing ammonium hydroxide was also prepared. The two microemulsions containing cerium nitrate and ammonium hydroxide were then mixed and stirred until a colloidal suspension was formed. The synthesized nanoparticles were separated by centrifugation and subsequently washed with methyl alcohol, deionized water, and acetone. Afterward, they were dried using freeze-drying and vacuum-drying methods. The obtained CeO_2_ NPs had a size distribution ranging from 2 to 6 nm [86].

Zhang et al. investigated the effects of the annealing temperature on the CeO_2_ nanocrystals prepared by a microemulsion method. The findings indicated that at 623 K as the annealing temperature, numerous spherical-shaped aggregates were observed, with a size of approximately 65 nm. When the annealing temperature was increased up to 873 K, CeO_2_ nanocrystals with a diameter of 6–8 nm appeared. Additionally, it was observed that as the annealing temperature was increased, there was a change in the phase structure from triclinic to cubic. Furthermore, the valence state of the cerium ions changed from Ce^3+^ to Ce^4+^. It is also noteworthy to mention that CeO_2_ nanocrystals were formed when the annealing temperature was above 773 K [87].

### 4.8 Other methods

Aside from the above-mentioned commonly used methods, there are other methods that could be used for the synthesis of CeO_2_ NPs. Surfactant-mediated precipitation technique in an acetone/water mixed solvent system [88], poly(vinylpyrrolidone)-assisted hydrothermal process [89], supercritical and subcritical solvothermal methods [90,91], microwave-assisted heating technique [92], microwave-mediated hydrothermal and microwave-mediated solvothermal methods [93] have all been proposed by researchers for the synthesis of nanoscale CeO_2_ in different shapes and sizes. It is important to note that the choice of a synthesis method depends on several factors, such as the desired particle size and shape, the intended application, and the availability of resources and equipment. Each method has its own advantages and limitations, which should be carefully considered when selecting a synthesis method.

## 5 Biosynthesis of CeO_2_ NPs

The concept of green synthesis is centered around the use of environmentally friendly and sustainable methods for the synthesis of compounds and chemicals, including nanoparticles [94]. Many studies have focused on the green synthesis of CeO_2_ NPs using different sources. The green synthesis of CeO_2_ NPs is a cost-effective, eco-friendly, and safe alternative to the traditional chemical synthesis methods. The biosynthesis of CeO_2_ NPs involves the use of natural plant extracts or other biological agents for the reduction of cerium ions to cerium oxide [18,95]. In this section, the biosynthesis of CeO_2_ NPs using plants, microbes, and food products will be discussed.

### 5.1 Synthesis from plant sources

Plants seem to be the most efficient source for the green synthesis of nanoparticles because of many advantages, such as their safe nature and abundance. They could be exploited as a rich source of reducing and stabilizing agents for the CeO_2_ NPs synthesis due to their high biocompatibility and cost-effectiveness. Different parts of plants (e.g., stem, flower, and leaves) have been used for this purpose; however, most of the studies have been performed on leaf extracts, which are rich in metabolites [95,96].

Various phytochemicals/metabolites (e.g., phenols, ketones, ascorbic acid, and carboxylic acids) have been employed as reducing and stabilizing agents. Synthesis of CeO_2_ NPs from plant sources is performed by a simple method in which a mixture of bulk metal salt and plant extract is provided, and the reaction proceeds in minutes to a few hours. Phytochemicals act as reducing and stabilizing agents. The synthesis of nanoparticles is primarily confirmed by solution color change. The physicochemical properties of the synthesized CeO_2_ NPs could be characterized by different imaging techniques and spectroscopic methods [96,97].

In this regard, Surendra and Roopan used *Moringa oleifera* peel as the stabilizing and reducing agent to synthesize CeO_2_ NPs. The synthesized nanoparticles were spherical with an average size of 45 nm, showing antibacterial activity against both Gram positive (*Staphylococcus aureus*) and Gram negative (*Escherichia coli*) bacteria [98]. Arumugam et al. used the leaf extract of *Gloriosa superba* to synthesize CeO_2_ NPs through a single-step process [99]. In a recent study, Eka Putri et al. [100] used *Moringa oleifera* leaf extract to synthesize CeO_2_ NPs through a simple and rapid green precipitation method. For this purpose, cerium nitrate hexahydrate (3.72 g) was added to the *Moringa oleifera* leaf extract (50 ml). The solution was stirred at 80 °C for 120 min, and the resulting solid was calcined at 600 °C for 120 min. The reported average size of the CeO_2_ NPs was 17 nm, which was comparable with that of CeO_2_ NPs synthesized using the leaf extracts of other plants such as *Origanum majorana* (20 nm) [101], *Datura metel* (18.78 nm) [102], and *Olea europaea* (24 nm) [103]. The synthesized CeO_2_ NPs showed high antibacterial activity against *S. aureus*, *E. coli*, and *P. aeruginosa*, as well as antifungal activity against *C. albicans* and *A. fumigatus*. Until now, the leaf extracts of a variety of plants such as *Caccinia macranthera* [104], *Artabotrys hexapetalus* [105], *Azadirachta indica* [106], *Prosopis farcta* [107], *Acalypha indica* [108], etc. have been used for the synthesis of CeO_2_ NPs with suitable physicochemical and biological properties for different biomedical applications.

Aside from the above-mentioned leaf extracts, other parts of plants have also been used for the synthesis of CeO_2_ NPs. In this regard, Elahi et al. prepared CeO_2_ NPs from Ce(NO_3_)_3_ and seeds extract of *Salvia macrosiphon Boiss* to investigate their photocatalytic properties. The results showed the effective degradation of Rhodamine B dye [109]. Flower extract of *Calotropis procera* has also been used to synthesize spherical CeO_2_ NPs with a particle size of 21 nm, which showed antibacterial properties and high effectiveness in the photocatalytic degradation of methyl orange dye [110]. Miri et al. used *Ziziphus jujube* fruit to synthesize CeO_2_ NPs (18–25 nm). The nanoparticles demonstrated excellent ultraviolet protection and sunscreen physical absorption [111]. In a study by Altaf et al. CeO_2_ NPs were synthesized using cerium(III) nitrate solution and the aqueous extract of *Acorus calamus* rhizome. The nanoparticles showed antibacterial properties against *S. aureus*, *P. aeruginosa*, and *E. coli* [112]. *Aloe barbadensis* miller gel [113], *Hibiscus sabdariffa* flower [114], *Moringa oleifera* peel [98], *Hyphaene thebaica* fruit [115], *citrus limon* peel [116], and *Linum usitatissimum* L. seeds [117] have also been used for the green synthesis of CeO_2_ NPs.

### 5.2 Synthesis from microorganisms

Microorganisms are also rich in secondary metabolites, making them a potential source for the green synthesis of CeO_2_ NPs. In recent years, there have been many reports on the synthesis of CeO_2_ NPs with different shapes and a wide range of sizes from microorganisms. Microbial metabolites have a critical role as reducing and stabilizing agents. Green synthesis of CeO_2_ NPs from microorganisms is simple, cost-effective, and ecologically-friendly [18,95]. Some studies have clearly described the suitability and potential of using fungi for the green synthesis of CeO_2_ NPs. In 2013, Khan et al. reported the green synthesis of CeO_2_ NPs using the thermophilic fungus *Humicola* sp. The nanoparticles were spherical in shape and had an average diameter of 16 nm [118].

The microorganism *Aspergillus niger* is a haploid fungus with industrial applications, including the production of extracellular enzymes and citric acid [[119], [120], [121]]. It is also used in studies on biotransformation and waste management [122,123]. Gopinath et al. used *Aspergillus niger* culture filtrate to synthesize antibacterial CeO_2_ NPs with particle sizes ranging from 5 to 20 nm. For this purpose, after the incubation of *Aspergillus niger* and the production of fungal spores, the culture medium was filtered. Cerium(III) chloride heptahydrate (CeCl_3_·7H_2_O) was added to the filtrate to form a precipitate, which was then calcined at 350 and 400 °C to produce CeO_2_ NPs. The synthesized CeO_2_ NPs exhibited antibacterial activity against *Proteus vulgaris*, *E. coli*, *Streptococcus pneumonia*, and *Bacillus subtilis* [124]. Munusamy et al. used a similar protocol to synthesize CeO_2_ NPs from the filtrate of *Curvularia lunata* culture media. The synthesized CeO_2_ NPs had a cubic structure with particle sizes ranging from 5 to 20 nm, displaying antibacterial activity against three Gram-negative (*Pseudomonas aeruginosa*, *Proteus vulgaris*, and *Klebsiella pneumoniae*) and three Gram-positive (*Streptococcus pneumoniae*, *Bacillus subtilis*, and *S. aureus*) bacteria [125]. In another study, the filtrate of *Fusarium solani* species culture media was used for the biosynthesis of spherical-shaped CeO_2_ NPs with an average size of 24.5 nm. The formation of CeO_2_ NPs was due to the presence of bio-organic compounds in *Fusarium solani* culture media. The nanoparticles exhibited good antibacterial properties against *P. aeruginosa*, *K. pnemoniae*, *S. aureus*, and *E. coli*. They also effectively inhibited the formation of bacterial biofilms [126].

Biosynthesis of CeO_2_ NPs using bacteria has also been investigated. Krishnaveni et al. reported the biosynthesis of spherical-shaped CeO_2_ NPs using the extracellular supernatant of *Bacillus subtilis*. The nanoparticles' crystalline nature and their average crystallite size of about 8 nm were confirmed by XRD analysis. The biosynthesized CeO_2_ NPs showed remarkable antioxidant capacity according to DPPH and reducing power assays. The authors suggested the potential use of these nanoparticles in the treatment of diseases associated with oxidative stress, such as rheumatoid arthritis, cardiovascular diseases, Parkinson's disease, atherosclerosis, and cancer [127].

### 5.3 Synthesis from food products

Aside from the aforementioned sources, food products have also been used in the cost-effective and environmentally-friendly synthesis of CeO_2_ NPs. In this regard, the starch-based synthesis of CeO_2_ NPs for biomedical applications has been reported in the literature. Darroudi et al. synthesized CeO_2_ NPs with a mean diameter of about 6 nm using a starch-mediated sol-gel method [128]. In another study, cassava starch was used as a chelating agent to synthesize CeO_2_ NPs. Ferreira et al. synthesized CeO_2_ nanoparticles by adding a Ce(NO_3_)_3_·6H_2_O solution to a cassava starch solution at 25 °C. The mixture was then stirred for 1h at 70 °C to complete the gelatinization of starch. The obtained gel was kept at 100 °C overnight and subsequently calcinated at temperatures ranging from 200 °C to 500 °C. By increasing the calcination temperature from 200 °C to 500 °C, the average crystallite size of the nanoparticles was increased from 8.11 nm to 12.69 nm [129].

Using cerium(III) acetate hydrate and ovalbumin, the major protein component of egg white, Maensiri et al. synthesized plate-like CeO_2_ NPs with particle sizes ranging from 6 to 30 nm. This study also demonstrated that the size of nanoparticles was increased with raising the calcination temperature [130]. In another study, egg white and cerium(III) nitrate hexahydrate were used to synthesize spherical-shaped CeO_2_ NPs, which demonstrated excellent antibacterial properties against *S. aureus* and *E. coli* [131].

Honey is another food product used as a biological source for the synthesis of nanoscale CeO_2_. In a recent study, pine honey, blossom honey, and chestnut honey were used for the environmentally friendly and cost-effective green synthesis of CeO_2_ NPs with average particle sizes of 3.02, 2.61, and 1.23, respectively. The nanoparticles showed high antioxidant activities according to the results of the DPPH (2,2-diphenyl-1-picryl-hydrazyl-hydrate) assay [132]. Darroudi et al. reported the successful synthesis of CeO_2_ NPs using cerium(III) nitrate hexahydrate and honey. Cerium cations were converted into CeO_2_ NPs through a sol–gel process in aqueous honey solutions [133].

Reddy Yadav et al. demonstrated that watermelon juice could be used to synthesize CeO_2_ NPs with photocatalytic activity and antibacterial properties against *Klebsiella aerogenes* and *S. aureus* [134].

Overall, the biosynthesis methods discussed in this subsection offer sustainable and eco-friendly approaches to synthesize CeO_2_ NPs for potential use in various biomedical applications.

## 6 Biological properties of CeO_2_ NPs for wound healing

CeO_2_ NPs have been found to possess several biological properties, making them suitable for a wide range of biomedical applications. CeO_2_ NPs have been shown to promote wound healing by reducing inflammation, oxidative stress, and the risk of infection while also promoting angiogenesis during the wound healing process. These properties make CeO_2_ NPs a promising candidate for wound healing applications.

### 6.1 Antioxidant and anti-inflammatory properties

Reactive oxygen species (ROS), which are chemically reactive molecules that contain oxygen, are generated as a by-product of normal cellular metabolism. ROS can be produced by various cellular processes, such as mitochondrial respiration, inflammation, and exposure to environmental stressors such as radiation and pollution [135]. While some ROS play important roles in cellular signaling and defense against pathogens, excessive ROS production can cause oxidative stress and damage cellular components such as DNA, proteins, and lipids [[136], [137], [138]]. To prevent oxidative damage, cells have evolved various antioxidant systems to neutralize ROS [135]. Antioxidants neutralize ROS by donating electrons to them, thus preventing them from causing oxidative damage to cells and tissues [139]. A balance between ROS and antioxidants is essential for maintaining proper cellular function and homeostasis [140].

Up to now, different antioxidant agents have been utilized to control the levels of ROS, among which CeO_2_ NPs are considered one of the most promising candidates. CeO_2_ NPs have demonstrated a remarkable ability to mimic antioxidant enzymes. They have the potential to eliminate both ROS, such as superoxide anion radicals, and reactive nitrogen species (RNS), like nitric oxide radicals [141,142]. In a study conducted by Cheng et al., concentration-dependent antioxidant activity of CeO_2_ NPs was demonstrated using the DPPH radical-scavenging assay, superoxide dismutase (SOD) mimetic activity assay, and catalase mimetic activity assay. As the concentration of CeO_2_ NPs was increased from 1 μg/mL to 300 μg/mL, the ability to scavenge DPPH free radicals was enhanced. The SOD mimetic activity assay also showed an increase until the concentration of CeO_2_ NPs reached 600 μg/mL. According to the catalase mimetic activity assay results, an increased activity was observed before the concentration of CeO_2_ NPs reached 1000 μg/mL [143].

Excessive and prolonged ROS production can lead to oxidative stress, which can contribute to the progression of inflammation and tissue damage. Therefore, CeO_2_ NPs are considered anti-inflammatory agents due to their ability to scavenge ROS. Hirst et al. investigated the ability of CeO_2_ NPs to scavenge ROS and inhibit the production of the inflammatory mediator inducible nitric oxide synthase (iNOS) in J774A.1 murine macrophages. The study showed that internalization of CeO_2_ NPs occurred when J774A.1 cells were treated with a 10 μM concentration of CeO_2_ NPs for 24h. The results also indicated that CeO_2_ NPs were able to quench ROS and inhibit the production of iNOS in J774A.1 cells. These findings, thus, suggest that CeO_2_ NPs have potential therapeutic applications for the treatment of inflammatory diseases [144]. Gojova et al. synthesized CeO_2_ NPs with an average diameter of approximately 40 nm using spray flame synthesis. Human aortic endothelial cells were incubated with various concentrations of CeO_2_ NPs (ranging from 0.001 to 50 μg/mL) for 4h. After the incubation period, the mRNA levels of three inflammatory markers, namely, intercellular adhesion molecule 1 (ICAM-1), interleukin 8 (IL-8), and monocyte chemotactic protein-1 (MCP-1), were measured. The results did not demonstrate significant inflammatory reactions [145]. In addition, CeO_2_ NPs have been shown to have anti-inflammatory activity by inhibiting the activity of inflammatory pathways. Domala et al. conducted a study to explore the effect of CeO_2_ NPs on psoriasis. The researchers used imiquimod to induce psoriasis in Balb/c mice; then they applied CeO_2_ NPs topically, intraperitoneally, and subcutaneously, once daily as a treatment. They found that the use of CeO_2_ NPs significantly decreased splenic hypertrophy, psoriasis severity, and lipid peroxidation. Furthermore, the expression of various inflammatory and proliferation markers, such as IL-17, IL-22, IL-23, Ki-67, NF-κB, COX-2, and GSK3, was reduced. The study concluded that CeO_2_ NPs had an antipsoriatic effect by inhibiting the Th-cell mediated IL-17/IL-23 immune axis and downregulating other crucial inflammatory proteins [146].

### 6.2 Antibacterial properties

Cerium has been found to be a promising and long-lasting biocide for preventing bacterial infections. Its unique antibacterial mechanism and higher safety to human cells make it an appropriate candidate for various biomedical applications compared to other metal ions. Therefore, cerium and cerium oxide-based antimicrobials have gained significant attention with various studies reporting different designs of cerium-related nanomaterials against pathogenic microorganisms. The primary antibacterial mechanism of CeO_2_ NPs is due to its direct contact with bacterial membranes [147]. At first, the positively charged nanoparticles adsorb the negatively charged membranes of the both Gram-negative and Gram-positive bacteria through electrostatic interactions, resulting in their adsorption onto the bacterial surface. This adsorption prevents the nanoparticles from penetrating the bacterial membrane and allows them to persist on the bacterial surface for a prolonged period. These nanoparticles subsequently alter the viscosity of the membrane, disrupt the function of certain ionic pumps, and significantly impact the exchange of transport between the bacterial cell and the environment, ultimately impeding bacterial growth [148,149]. Secondly, CeO_2_ NPs could have a detrimental effect on proteins once they have been adsorbed onto the outer membrane of bacterial cells. One consequence of this interaction could be the release of cerium ions, which may disrupt the flow of electrons and respiration of bacteria [150]. Additionally, these released ions may react with thiol groups (-SH) or be absorbed to transporters and/or porins, which could impede the transportation of nutrients [147,151]. Moreover, the physical characteristics of CeO_2_ NPs, such as their irregular shapes and rough edges, may cause physical damage to bacterial membranes, especially in the case of Gram-positive bacteria [152].

**Fig. 3:**
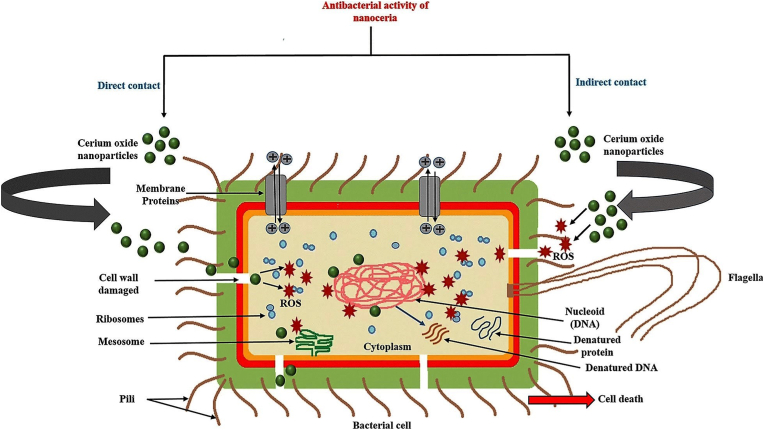
The direct and indirect interactions of CeO_2_ NPs with bacterial cell. The direct interaction results in the damage to the cell wall and penetration of the nanoparticles into the bacteria. The generation of ROS causes damage to DNA and proteins. In the indirect interaction, ROS are produced outside the bacterial cell and then enter the cell through the cell membrane, where they can cause damage to DNA and proteins. Reprinted from [52].

In some biological systems, the primary mechanism of nanomaterial toxicity has been attributed to the generation of ROS, which causes oxidative stress. The chemical degradation of various organic components in microorganisms can be caused by ROS, resulting in significant damage to bacteria [147,152]. Another destructive effect of CeO_2_ NPs on bacterial cells is the elevation of ROS levels, which can lead to damages to DNA, RNA, proteins, lipids, and polysaccharides [149]. Zholobak et al. explained how CeO_2_ NPs could indirectly interact with microorganisms encapsulated in polysaccharides or form biofilms, where CeO_2_ NPs do not have direct access to the cell membrane. In this particular mechanism, the cerium ions resulting from the dissolution of nanoparticles and the ROS formed on the surface of the particles play a crucial role. In this type of interaction, ROS are produced outside the bacterial cell and then enter the cell through the cell membrane, where they can damage bacterial cells [153]. It has also been reported that CeO_2_ NPs have peroxidase mimetic activity, which can lead to the generation of ROS [154,155]. Fig. 3 depicts the direct and indirect interactions of CeO_2_ NPs with bacterial cells.

### 6.3 Angiogenic properties

As previously stated, angiogenesis is the process by which new blood vessels are formed from pre-existing vessels. It is a physiological process that occurs during development and wound healing, as well as in pathological conditions such as cancer. During angiogenesis, endothelial cells that line the existing blood vessels begin to proliferate and migrate toward the site where new blood vessels are needed. These cells then form a tube-like structure, which becomes a new blood vessel. During the wound healing process, as the wound heals, the newly formed blood vessels provide oxygen and nutrients to the cells involved in the tissue repair, such as fibroblasts and immune cells. The blood vessels also help remove waste products and toxins from the site of injury. In recent years, there have been many efforts to promote angiogenesis in the injured tissue using various strategies in order to accelerate the healing process. The use of nanomaterials has opened up significant opportunities for tissue engineering, particularly in the area of angiogenesis, which has been a major challenge in the field of regenerative medicine [5,15].

Based on experimental evidence, it is suggested that CeO_2_ NPs have the capability to both induce and inhibit the formation of new blood vessels [141,149]. In a study conducted by Das et al. it was shown that CeO_2_ NPs could induce angiogenesis by regulating the intracellular oxygen environment. CeO_2_ NPs having a diameter range of 3–5 nm with a high surface Ce^3+^/Ce^4+^ ratio had the ability to enhance the formation of vascular endothelial cell tubes *in vitro*. They also stimulated the robust growth of blood vessels in a chick chorioallantoic membrane (CAM) assay. It was found that the angiogenic activity of the nanoparticles was dependent on their material properties. Smaller nanoparticles (≤15 nm) and higher surface Ce^3+^/Ce^4+^ ratios resulted in robust induction of blood vessel formation. The researchers also observed a correlation between the nanoparticles' ability to reduce intracellular oxygen levels and their capacity to induce angiogenesis. This reduction in oxygen levels was found to transiently induce the translocation of hypoxia-inducing factor 1α (HIF-1α) to the nucleus, which, in turn, stimulates the expression of several proteins involved in angiogenesis [156]. The ability of CeO_2_ NPs to regulate angiogenesis has encouraged researchers to further delve into the impact of these particles on the process of wound healing. In this regard, Chigurupati et al. demonstrated the accelerated healing of full-thickness dermal wounds in male C57BL/6 mice following the topical application of water-suspended CeO_2_ NPs. This was achieved by stimulating the proliferation and migration of fibroblasts, keratinocytes, and vascular endothelial cells. The results also showed that CeO_2_ NPs significantly increased the number of blood vessels in the healing wounds. Furthermore, CeO_2_ NPs aided the regeneration process by reducing oxidative stress [157].

It should be noted that CeO_2_ NPs can also have anti-angiogenic properties, depending on the microenvironment. This may be attributed to their pH-dependent activity. CeO_2_ NPs tend to accumulate hydrogen peroxide (H_2_O_2_) in acidic environments while they scavenge H_2_O_2_ at physiological pH. This means that in a tumor environment with an acidic pH, CeO_2_ NPs may cause the formation of a significant amount of H_2_O_2_, which could potentially impede tumor growth and inhibit the formation of blood vessels [141].

Along with the aforementioned biological properties of CeO_2_ NPs that make them suitable for wound healing applications, there have been efforts to modify CeO_2_ NPs to enhance their effectiveness in promoting multiple stages of the wound healing process. In this regard, Cheng et al. designed arginine-loaded ceria-graphene nanocomposites in which ceria nanoparticles and graphene were linked by N-hydroxysuccinimide (NHS) ester. At the inflammatory phase of the wound healing process, the prepared nanocomposites could effectively generate ROS and kill bacteria under white light irradiation due to their efficient electron-hole separation between ceria nanoparticles and graphene. At the proliferation phase, ceria nanoparticles could be detached and taken up by cells to scarify intracellular ROS and promote cell proliferation, while the separated graphene could act as a scaffold to promote fibroblast migration to the wound site [158]. In a study done by Ma et al. hollow CeO_2_ NPs with a porous shell and rough surface were prepared and loaded with L-arginine to promote multiple stages of the wound healing process. During the hemostasis stage, the modified CeO_2_ NPs could serve as a nanobridge within the tissue to rapidly halt bleeding. During the inflammatory phase, these nanoparticles could generate ROS to kill bacteria under simulated sunlight irradiation, preventing bacterial infection. At the proliferation stage, the modified CeO_2_ NPs could capture excessive ROS generated at the wound site due to their SOD and catalase activities. Additionally, the released L-arginine could be converted into nitric oxide by iNOS in macrophages, promoting cell proliferation [159].

## 7 Scaffolds and dressings containing CeO_2_ NPs for wound healing applications

Designing and developing multifunctional scaffolds and dressings with optimal properties for skin tissue engineering and wound healing applications has been the focus of a wide range of studies in recent years. Several fabrication methods have been employed for this purpose to obtain constructs capable of mimicking natural tissue and providing cell support to facilitate skin tissue regeneration. Table 1 summarizes some of the fabrication techniques used for creating such constructs. It is important to note that each method has its own advantages and limitations that should be considered.

**Table 1:** Common methods for fabricating scaffolds/dressings for skin tissue engineering and wound healing applications.

Fabrication method	Short Description	Advantages	Limitations	References
Electrospinning	Using an electric field to produce fibers from a polymer solution	- Production of uniform nanofibers with high surface area to volume ratio - High porosity with interconnected pores - Ability to control fiber diameter - The use of a variety of polymers - ECM-like structure - Easy to perform (a simple fabrication method)	- Needs high-voltage equipment - May require toxic solvents - Can be challenging to control fiber alignment - Limitation on bioceramic loading into the polymer matrix	[[160], [161], [162], [163]]
Freeze-drying	Freezing a polymer solution and then removing the solvent by sublimation to create a porous scaffold	- Creates highly porous scaffolds - The use of a variety of polymers - Scalable - Relatively simple process	- Needs equipment (freeze dryer apparatus) - Time-consuming production - Limited pore size control - Can be difficult to control porosity - May require toxic solvents	[[164], [165], [166]]
Solvent casting	Casting a polymer solution into a mold, and then evaporating the solvent to create a solid scaffold	- Easy to perform (a simple fabrication method) - The use of a variety of polymers - Good mechanical properties - Requires relatively cheap equipment	- Limited control over scaffold architecture - Lacks reproducibility - Possibility for the retention of toxic solvent within the scaffold	[160,166,167]
Gas foaming	Using a gas to create pores in a polymer scaffold	- Solvent-free (Environmentally friendly) - Scalable	- Needs high pressure - Poor interconnectivity of pores - Crystalline polymers cannot be used	[160,166,168]
Phase separation	Inducing phase separation in a polymer solution to create a porous scaffold	- High porosity - Easy to perform (a simple fabrication method)	- May require using toxic solvents - Limited control over scaffold architecture - Limited control over pore sizes	[160,166]
3D printing	Fabricating a three-dimensional structure layer by layer using a computer-aided design (CAD) model.	- High precision - Can create complex shapes and geometries - Scalable - Exact control on morphology and size of the pores - Repeatable - Possible to load high level of bioceramic (more than 60 wt%) into the polymeric matrix	- Requires equipment (3D printer)	[[169], [170], [171], [172], [173]]

Electrospinning is a versatile and innovative technique used to produce fibers with diameters ranging from a few nanometers to several micrometers. In this process, the application of a high-voltage electric field to a polymer solution or melt leads to the formation of a jet, known as the Taylor cone, which is rapidly elongated and solidified into fibers [160,174]. Electrospinning has gained significant attention in recent years due to its potential applications in various fields, including biomedical applications. Electrospun nanofibrous scaffolds possess unique properties, such as high surface-to-volume ratio, nanoscale morphology, high porosity, and pore connectivity, making them suitable for delivering therapeutic agents for wound healing applications [160,175]. The incorporation of bioactive agents and nanomaterials into electrospun nanofibers has led to their use as delivery systems, where they can provide the sustained release of the therapeutic agents during the wound healing process. Additionally, electrospun nanofibers have been demonstrated to successfully mimic the natural ECM and support cell proliferation, migration, and differentiation [176,177].

**Fig. 4:**
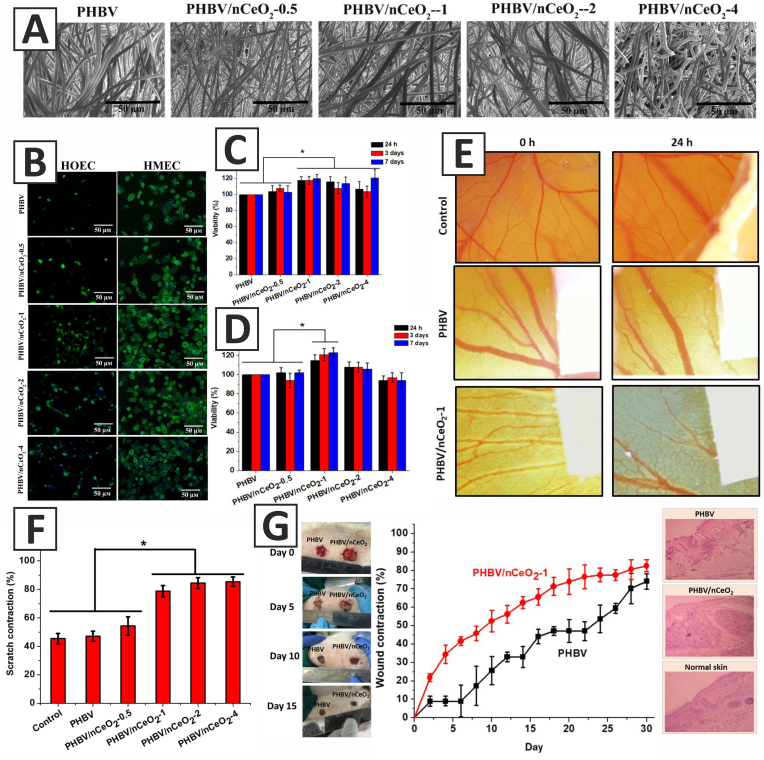
**A**) SEM images of the PHBV and CeO_2_ NPs-containing PHBV membranes. **B**) Adhesion of human oral epithelial cells (HOEC) and human mammary epithelial cells (HMEC) to the membranes after 3 days of cell culture_ DAPI-phalloidin staining. **C**) Viability of HMEC cultured on the membranes. **D**) Viability of HOEC cultured on the membranes. **E**) CAM angiogenesis assay showing angiogenic potential of PHBV/nCeO_2_-1. **F**) The effects of PHBV and CeO_2_ NPs-containing PHBV membranes on wound contraction in scratch assay. **G**) The effects of PHBV and PHBV/nCeO_2_-1 membranes on the healing of full-thickness excision wounds in diabetic rats. (PHBV: poly(3-hydroxybutyrate-*co*-3-hydroxyvalerate), PHBV/nCeO_2_-0.5, PHBV/nCeO_2_-1, PHBV/nCeO_2_-2, and PHBV/nCeO_2_-4: PHBV membrane containing 0.5, 1, 2, and 4% w/w CeO_2_ NPs, respectively). Adapted with permission from Ref. [178]. Copyright 2019 American Chemical Society.

Augustine et al. also developed electrospun poly(3-hydroxybutyrate-*co*-3-hydroxyvalerate) (PHBV) membranes containing different concentrations of CeO_2_ NPs (0.5%, 1%, 2%, or 4% w/w) for the treatment of full-thickness excision wounds in diabetic male Sprague Dawley rats (Fig. 4 (A)). The nanofibrous scaffolds exhibited high porosity, which could support cell migration and provide enough space for the ECM production during wound healing. The results of the mechanical tests also revealed that PHBV membranes incorporated with 1% w/w CeO_2_ NPs (PHBV/nCeO_2_-1) had the highest tensile strength (4.38 ± 0.36 MPa) and Young's modulus (11.18 ± 3.14 MPa), which were reported to be appropriate for wound healing applications. *In vitro* studies demonstrated the higher adhesion of human oral epithelial cells (HOEC) and human mammary epithelial cells (HMEC) on PHBV scaffolds loaded with CeO_2_ NPs (Fig. 4 (B)). MTT tests showed the high biocompatibility of the CeO_2_ NPs-incorporated membranes, among which the PHBV/nCeO_2_-1 membrane displayed the highest biocompatibility and significantly increased the proliferation of both HOEC and HMEC, as compared to the PHBV membrane (Fig. 4 (C and D)). In addition, the results of the scratch assay showed that PHBV membranes containing 1, 2, or 4% w/w CeO_2_ NPs (PHBV/nCeO_2_-1, PHBV/nCeO_2_-2, and PHBV/nCeO_2_-4, respectively) could significantly enhance the migration of HaCaT keratinocytes *in vitro* (Fig. 4 (F)). Based on the physicochemical properties of the prepared membranes and the *in vitro* tests performed, PHBV/nCeO_2_-1 membrane was chosen for further evaluations. The results of the CAM assay showed the high ability of CeO_2_ NPs for the induction of angiogenesis, as the PHBV/nCeO_2_-1 membrane significantly increased the formation of blood vessels, compared to the PHBV membrane (Fig. 4 (E)). PHBV/nCeO_2_-1 membrane could also considerably enhance the healing of full-thickness excision wounds in diabetic rats (Fig. 4 (G)) [178].

Solvent casting is a commonly used method for fabricating scaffolds for biomedical applications. In this method, a polymer is dissolved in a solvent, and the resulting solution is cast into a mold to obtain the desired shape. Once the solvent evaporates, a solid structure is formed [179]. The choice of material depends on the desired properties of the scaffold and the intended biomedical application. Scaffolds developed by the solvent casting method have shown great potential in promoting wound healing by providing a supportive structure for cell growth and tissue regeneration [180,181]. Introducing nanoparticles into the structure of such scaffolds can improve mechanical and biological properties of the scaffolds, enhancing their therapeutic efficacy. Nanoparticles can be incorporated into the scaffold materials by adding them to the polymer solution prior to casting [[182], [183], [184]]. It is noteworthy to mention that the type of nanoparticles and the scaffold material are among the primary factors that should be considered for determining the optimal concentration of the nanoparticles.

Kalaycıoğlu et al. used the solvent casting technique to prepare polysaccharide-based films composed of chitosan and cellulose acetate loaded with CeO_2_ NPs. Formic acid at concentrations of 2% (w/v) and 5% (w/v) was used to dissolve chitosan and cellulose acetate, respectively. The prepared films were then characterized in terms of their physical and mechanical properties. The antibacterial activity of the films was also evaluated. The results showed that the incorporation of CeO_2_ NPs increased the thermal stability of the films. The films loaded with CeO_2_ NPs also exhibited significantly higher Young's modulus, ultimate tensile strength, and fracture strength. The incorporation of CeO_2_ NPs significantly affected the physical properties of the films by decreasing the swelling ratio, water solubility, and moisture content, while increasing the water vapor transmission rate and water vapor permeability. The chitosan/cellulose acetate films showed antibacterial activity against *E. coli* and *S. aureus* bacteria. The incorporation of CeO_2_ NPs significantly improved the antibacterial activity of the films. The researchers proposed that chitosan/acetate films loaded with CeO_2_ NPs could be suitable as potential constructs for wound healing applications [183].

The process of 3D printing involves the fabrication of physical objects from digital models through the deposition of successive layers of material. Over the past few decades, the use of 3D printing techniques has expanded beyond its conventional use in industrial manufacturing and prototyping, and it has become more prevalent in biomedical applications. 3D printing technology has found new and valuable applications in the fields of medicine and biology. The ability to accurately place materials in three dimensions has proven to be particularly beneficial in areas such as surgery and tissue engineering [[185], [186], [187]]. When it comes to skin tissue engineering and wound healing applications, 3D printing can be used to design and fabricate advanced constructs with specific features that can provide a suitable environment for cells [169,188]. Moreover, drugs, growth factors, and other bioactive agents can be incorporated into the constructs, which can provide a sustained/controlled release system to enhance the efficacy of the treatment [189,190].

**Fig. 5:**
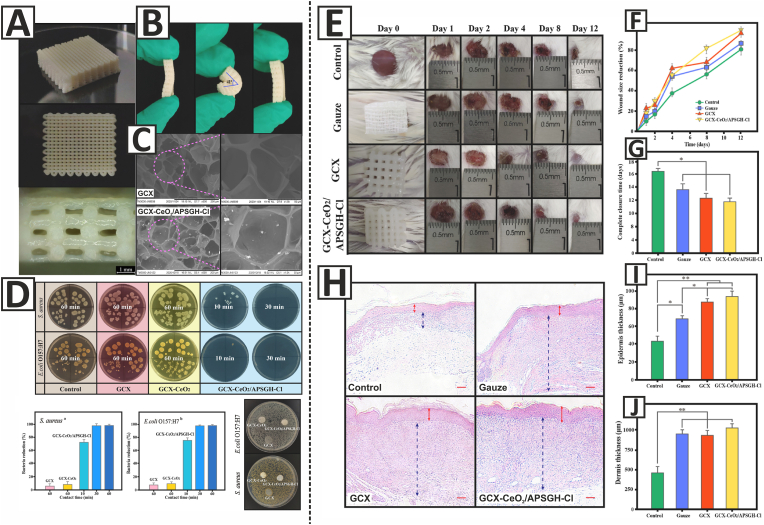
**A**) Side view, top view, and cross-section of the 3D-printed GelMA-based dressings containing 8 wt% CMC and 2 wt% xanthan gum. **B**) The suitable elasticity of the GCX dressing. **C**) SEM images of GCX and GCX-CeO_2_/APSGH-Cl dressings. **D**) Antibacterial activity of GCX, GCX-CeO_2_, and GCX-CeO_2_/APSGH-Cl dressings. **E**) The effects of gauze, GCX, and GCX-CeO_2_/APSGH-Cl dressings on the full-thickness wounds. Untreated wounds were considered as the control group. **F**) Wound closure rate after 12 days of treatment. **G**) Complete closure time of the wounds in various treatments. **H**) H&E-stained sections of the skin tissues after 8 days of treatment. The epidermis and the dermis are indicated with red and blue arrows, respectively. The scale bar is 50 μm. **I**&**J**) The thickness of epidermis and dermis after 8 days of treatment. (GCX: GelMA/CMC/xanthan gum, GCX-CeO_2_: GelMA/CMC/xanthan gum/CeO_2_ NPs, and GCX-CeO_2_/APSGH-Cl: GelMA/CMC/xanthan gum/N-halamine-modified CeO_2_ NPs). Adapted with permission from Elsevier [191]. (For interpretation of the references to colour in this figure legend, the reader is referred to the Web version of this article.)

In this regard, Yang et al. developed 3D-printed GelMA-based hydrogel dressings containing N-halamine-modified CeO_2_ NPs for the treatment of full-thickness skin wounds (Fig. 5). GelMA was used as the main component of the dressings, while Irgacure 2959 was applied as a photoinitiator for crosslinking the hydrogels under UV radiation. Xanthan gum and carboxymethylcellulose sodium (CMC) were used to adjust the printability of the dressings. CeO_2_ NPs and N-halamine-modified CeO_2_ NPs were also added to the ink formulations. The 3D-printed dressings exhibited excellent elasticity to meet the application demands of clinical wound treatment in terms of mechanical properties. The wound dressings also showed high swelling ratio values, as compared to common dressings, demonstrating high capacity of the prepared dressings for the absorption of wound exudate. SEM images showed that the presence of N-halamine-modified CeO_2_ NPs in the structure of the dressings could not significantly affect the morphology of the GelMA-based constructs. The dressings containing N-halamine-modified CeO_2_ NPs (GCX-CeO_2_/APSGH-Cl) exhibited high antibacterial activity against *S. aureus* and *E. coli*, as compared with dressings containing pure CeO_2_ NPs (GCX-CeO_2_) and CeO_2_ NPs-free dressings (GCX). According to the CCK-8 assay results, the survival rate of human pulmonary epithelial cells (A549 cells) was 91% after incubation with the leaching solution of GCX-CeO_2_/APSGH-Cl, thus indicating the *in vitro* cytocompatibility of the dressings’ materials. This could be attributed to the excellent biocompatibility of GelMA, CMC, and xanthan gum, as well as the good stability of N-halamine-modified CeO_2_ NPs. The hemolysis ratios of GCX and GCX-CeO_2_/APSGH-Cl were less than 2% even at a high concentration of 1500 μgmL^−1^, showing their high hemocompatibility. BALB/c mice with full-thickness excision wounds were used to evaluate the efficacy of the prepared dressings in enhancing the healing process *in vivo*. GCX and GCX-CeO_2_/APSGH-Cl dressings enhanced re-epithelialization and accelerated the wound healing process. As the conclusion, the outcomes showed the potential of 3D-printed GelMA-based dressings containing N-halamine-modified CeO_2_ NPs for wound care [191].

**Fig. 6:**
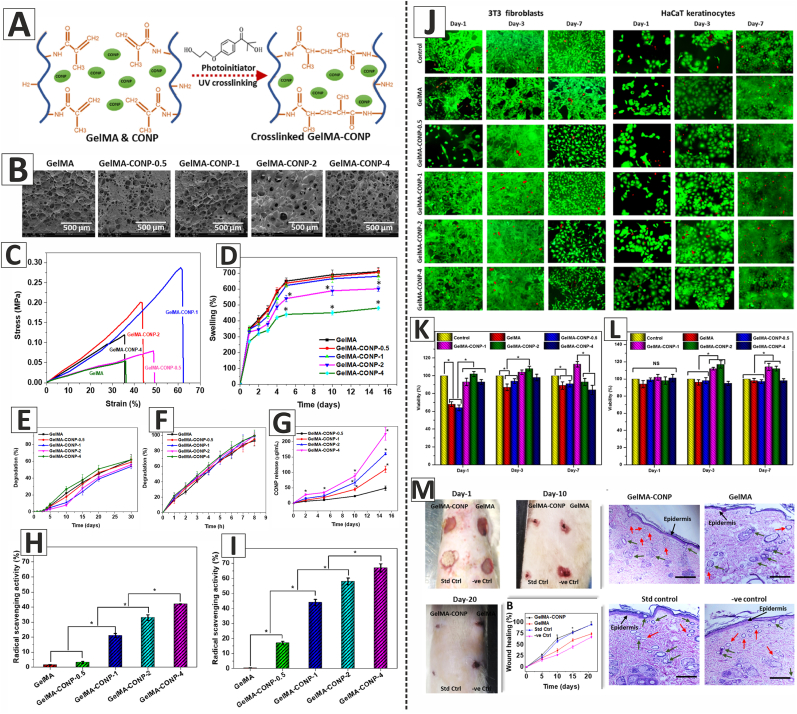
**A**) Preparation of the UV-crosslinked GelMA/CeO_2_ NPs (GelMA-CONP) hydrogels using 2-hydroxy-4′-(2-hydroxyethoxy)-2-methylpropiophenone (Irgacure 2959) as the photoinitiator. **B**) SEM images of the GelMA and GelMA-CONP hydrogels. **C**) Mechanical properties of the hydrogels. **D**) Swelling ratio of the hydrogels. **E**) Degradation rate of the hydrogels in PBS. **F**) Degradation rate of the hydrogels in the presence of collagenase enzyme. **G**) CeO_2_ NPs release from the GelMA-CONP hydrogels. **H**&**I**) DPPH and ABTS assays showing the free radical scavenging activity of the hydrogels in a CeO_2_ NPs concentration-dependent manner. **J**) Live/Dead assay showing adhesion and proliferation of 3T3 fibroblasts and HaCaT keratinocytes on the hydrogels. **K**&**L**) Viability of 3T3 fibroblasts and HaCaT keratinocytes cultured on the hydrogels. **M**) The effects of GelMA and GelMA-CONP hydrogels on the healing of full-thickness excision wounds in diabetic rats. Healing wounds treated with Puracol Plus Ag^+^ wound dressing was considered as the standard control (Std Ctrl), and untreated wounds were taken as the negative control (-ve Ctrl). (GelMA: gelatin methacryloyl, GelMA-CONP-0.5, GelMA-CONP-1, GelMA-CONP-2, and GelMA-CONP-4: GelMA hydrogels containing 0.5, 1, 2, and 4% w/w CeO_2_ NPs, respectively). Adapted with permission from Ref. [21]. Copyright 2020 American Chemical Society.

Aside from the above-mentioned techniques, other methods have also been used to fabricate scaffolds and dressings loaded with CeO_2_ NPs for wound healing applications. In this regard, Augustine et al. developed UV-crosslinked gelatin methacryloyl (GelMA) hydrogels containing various concentrations of CeO_2_ NPs (0.5, 1, 2, or 4% w/w) to promote the healing of full-thickness excision wounds in diabetic male Sprague Dawley rats (Fig. 6). The freeze-dried hydrogels had a bulk porosity ranging from 68 to 74%, with pore sizes ranging from 40.7 to 266.5 μm, providing ample space for cell proliferation, nutrient exchange, and fluid transport. Notably, hydrogels incorporating CeO_2_ NPs exhibited higher ultimate tensile strength (UTS) and Young's modulus, as compared to the GelMA hydrogel. The prepared hydrogels demonstrated a high swelling ratio, which could be attributed to the hydrophilic nature of GelMA. This high water uptake capacity is a desirable property for the treatment of highly exudating chronic wounds. Additionally, the hydrogels exhibited an excellent degradation rate that ensured the sustained release of CeO_2_ NPs. The free radical scavenging activity of the hydrogels was assessed using the DPPH and ABTS assays, showing that the scavenging activity of the hydrogels depended on the CeO_2_ NPs concentration, with higher concentrations resulting in higher activity. The Live/Dead assay demonstrated adhesion and proliferation of 3T3 fibroblasts and HaCaT keratinocytes on the hydrogels. The MTT assay results showed that GelMA hydrogels containing 1% w/w CeO_2_ NPs (GelMA-CONP-1) had promising results and could enhance the viability of both 3T3 fibroblasts and HaCaT keratinocytes. Based on the physicochemical and mechanical properties of the hydrogels and the *in vitro* cell viability tests, GelMA-CONP-1 hydrogel was chosen for *in vivo* evaluations. GelMA-CONP-1 hydrogel promoted re-epithelialization and accelerated the healing of full-thickness excision wounds in diabetic rats. The higher rate of angiogenesis in the healing wounds treated with GelMA-CONP-1 hydrogel, as well as the free radical scavenging activity of the CeO_2_ NPs, could be considered as the primary reasons for the accelerated wound healing [21].

**Fig. 7:**
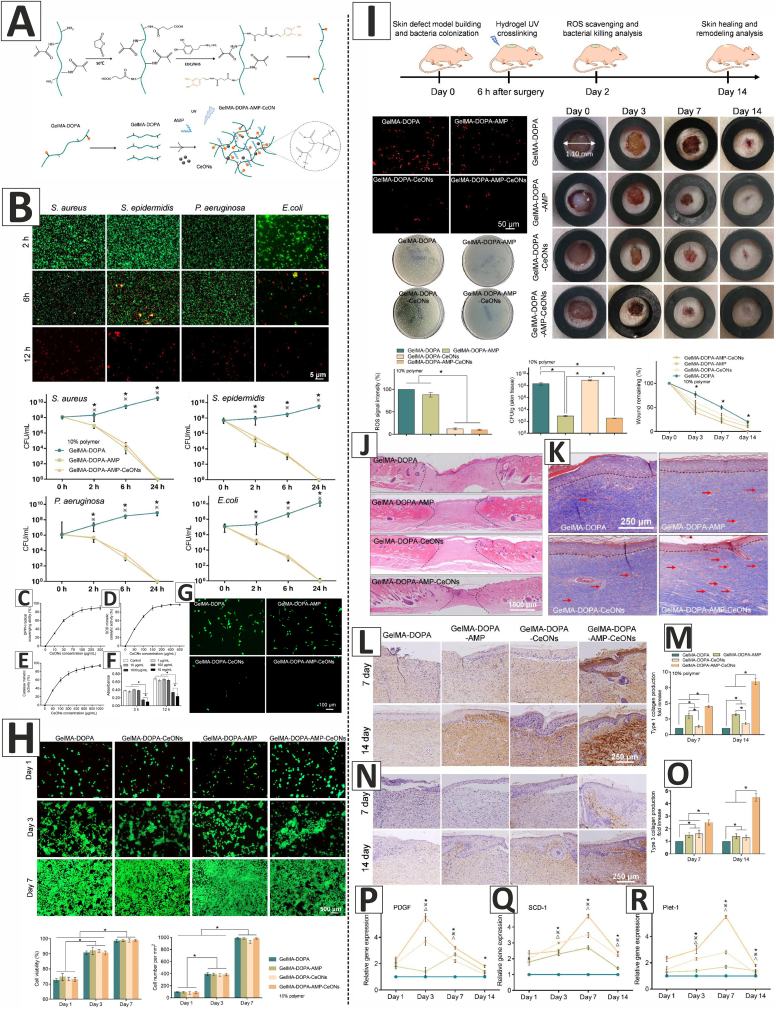
**A**) Schematic illustration of the preparation of GelMA/dopamine-based hydrogels loaded with CeO_2_ NPs and AMP (GelMA-DOPA-AMP-CeONs). **B**) Antibacterial properties of the prepared hydrogels, showing antibacterial properties of AMP-loaded GelMA-DOPA hydrogels against *S. aureus*, *S. epidermidis*, *P. aeruginosa*, and *E. coli* bacteria. **C**, **D**, & **E**) DPPH radical-scavenging assay, SOD-mimetic activity assay, and CAT-mimetic activity assay, displaying concentration-dependent antioxidant activity of CeO_2_ NPs. **F**) Viability of HaCaT cells assessed by CCK-8 test shows cytocompatibility of 100 μg/mL CeO_2_ NPs concentration. **G**) DCFH-DA assay showing reduced ROS signals in HaCaT cells cultured on the CeO_2_ NPs-loaded hydrogels. **H**) Live/dead staining showing the viability and proliferation of HaCaT cells cultured with the prepared GelMA-DOPA-based hydrogels. **I**) The effects of the GelMA/dopamine-based hydrogels on *S. aureus*-infected full-thickness wounds in SD rats. DHE staining demonstrating the ROS scavenging ability of the CeO_2_ NPs-loaded hydrogels *in vivo* on day 2 of the experiment. AMP-loaded hydrogels could almost completely ablate *S. aureus* bacteria on day 2. GelMA-DOPA-AMP-CeONs dressing showed the most promising results in accelerating the healing of *S. aureus-*infected wounds, as compared to other prepared GelMA/dopamine-based hydrogels. **J**) H&E staining shows decreased scar formation in *S. aureus*-infected wounds treated with GelMA-DOPA-AMP-CeONs hydrogels on day 14 of the experiment. **K**) Masson's trichrome staining shows the regenerated epithelium and newly formed blood vessels (red arrows) in different groups. **L**, **M**, **N**, & **O**) Immunohistochemistry (IHC) staining showing the deposition of collagen type I and III on days 7 and 14 of treatment. **P**, **Q**, & **R**) The expression levels of skin remodeling-associated genes, including SCD-1, LRIG-1, and PDGF-α analyzed by RT-PCR on days 1, 3, 7, and 14 of the treatment. Adapted with permission from Elsevier [143]. (For interpretation of the references to colour in this figure legend, the reader is referred to the Web version of this article.)

Cheng et al. designed, developed, and characterized sprayable GelMA/dopamine hydrogel dressings loaded with CeO_2_ NPs and an antimicrobial peptide (AMP). The dressings were aimed at enhancing the healing process of Sprague Dawley rats' full-thickness wounds infected with *S. aureus* due to the ROS scavenging properties of CeO_2_ NPs and the antibacterial properties of AMP (Fig. 7). To obtain the sprayable hydrogels, freeze-dried GelMA-dopamine was dissolved in deionized water. Irgacure 2959 photoinitiator was then added, followed by the addition of CeO_2_ NPs and AMP. The resulting mixture was transferred into a polydimethylsiloxane (PDMS) mold and exposed to UV radiation to obtain GelMA/dopamine/AMP/CeO_2_ NPs hydrogels (abbreviated as GelMA-DOPA-AMP-CeONs in this study). GelMA-DOPA-AMP and GelMA-DOPA-AMP-CeONs hydrogels exhibited excellent antibacterial activity against two Gram-negative (*P. aeruginosa* and *E. coli*) and two Gram-positive (*S. aureus* and *S. epidermidis*) bacteria. These two AMP-loaded hydrogels could rapidly ablate the four bacterial strains, mainly due to the presence of AMP. On the other hand, CeO_2_ NPs exhibited concentration-dependent antioxidant activity, as shown by the results of DPPH radical-scavenging assay, SOD mimetic activity assay, and catalase mimetic activity assay. Additionally, the CCK-8 assay results indicated that the viability of HaCaT cells was not compromised by hydrogels containing a concentration of 100 μg/mL of CeO_2_ NPs. Based on these findings, GelMA-DOPA-CeONs and GelMA-DOPA-AMP-CeONs containing 100 μg/mL of CeO_2_ NPs were used to investigate the intracellular ROS scavenging activity of CeO_2_ NPs-loaded hydrogels using dichlorodihydrofluorescein diacetate (DCFH-DA) assay. The HaCaT cells cultured on CeO_2_ NPs-loaded hydrogels showed significantly reduced ROS signals, as compared to the cells cultured on the CeO_2_ NPs-free hydrogels. The viability and proliferation of HaCaT cells cultured on GelMA/dopamine-based hydrogels were assessed using live/dead staining. The cells cultured on all of the prepared hydrogels showed high viability, with more than 90% viability after three days, thus indicating the cytocompatibility of the GelMA/dopamine-based hydrogels. The prepared hydrogels were used to treat *S. aureus*-infected full-thickness wounds in SD rats. Dihydroethidium (DHE) staining was used to detect ROS signals at the wound sites, demonstrating the ROS scavenging ability of CeO_2_ NPs-loaded hydrogels *in vivo*. Investigations on the *in vivo* antibacterial activity of the scaffolds also showed that the AMP-loaded hydrogels could almost completely eliminate *S. aureus* bacteria, as compared to AMP-free hydrogels. GelMA-DOPA-AMP-CeONs dressings significantly accelerated the healing of *S. aureus*-infected wounds. Histological analyses revealed decreased scar formation, increased deposition of collagen types I and III, as well as promoted regeneration of skin appendages in the tissues treated with GelMA-DOPA-AMP-CeONs hydrogels. Furthermore, the expression levels of skin remodeling-associated genes, including SCD-1, LRIG-1, and PDGF-α, were significantly increased, thus indicating the enhanced remodeling of the healing tissues [143].

To date, there have been several efforts to design and develop various CeO_2_ NPs-loaded scaffolds and dressings for skin tissue engineering and wound healing applications using different biomaterials and techniques. Table 2 summarizes some of the most important studies with promising outcomes over the past few years.

**Table 2:** Scaffolds/dressings containing CeO_2_ NPs for wound healing applications.

Biomaterials	CeO_2_ NPs preparation	Scaffold/wound dressing type	*In vitro* assay (Cell type)	*In vivo*/Animal model	Main outcomes	Refs
Chitosan/PVA/CeO_2_ NPs	CeO_2_ NPs synthesized using *Zingiber officinale* extract	Hydrogels prepared by a freeze-thaw technique	Live/Dead cell staining and PrestoBlue cell viability assay (Primary HDFs)	–	- Suitable physicochemical properties for wound healing applications - Incorporation of CeO_2_ NPs increased porosity and swelling ratio - No cytotoxicity to HDFs - Antibacterial activity against gram-positive MRSA	[192]
GelMA/CeO_2_ NPs	Synthesized using cerium nitrate (Ce(NO_3_)_3_·6H_2_O) as the precursor and gelatin as the stabilizing agent	UV-crosslinked hydrogel	Live/Dead cell staining and MTT assay (HaCaT keratinocytes and 3T3 fibroblasts)	Diabetic male SD rats with full-thickness excision wounds	- High porosity - Excellent exudate uptake capacity - Excellent free radical scavenging activity - Enhanced proliferation of 3T3 fibroblasts and HaCaT keratinocytes - Promoted re-epithelialization - Accelerated wound healing rate	[21]
PHBV/CeO_2_ NPs	Cerium nitrate (Ce(NO_3_)_3_·6H_2_O) was used as the precursor and gelatin as the stabilizing agent	Electrospun membrane	DAPI and phalloidin staining, crystal violet staining, MTT cell viability assay (HOECs and HMECs), scratch assay (HaCaT keratinocyte)	Diabetic male SD rats with full-thickness excision wounds	- High porosity - Increased tensile strength due to the presence of CeO_2_ NPs - Enhanced cell viability and adhesion of HOECs and HMECs - Promoted keratinocytes migration in scratch assay - Enhanced blood vessel formation in chicken CAM assay - Enhanced cell infiltration and granulation tissue formation - Enhanced diabetic wound healing	[178]
CBMA or SBMA/HEMA/miRNA146a-conjugated CeO_2_ NPs	CeO_2_ NPs synthesized using wet chemical method	Zwitterionic cryogels	CCK-8 assay (MC3T3 cells)	Diabetic female mice with full-thickness wounds	- Injectability and self-healing ability - No cytotoxicity - Sustained release of miRNA146a-conjugated CeO_2_ NPs - Accelerated the wound healing process - Enhanced biomechanical properties of skin - Increased expression of miR146a and type 1 collagen genes - Down-regulated pro-inflammatory cytokines IL-6 and CXCL2	[193]
PEO-CeO_2_ NPs-peppermint oil/graphene oxide	CeO_2_ NPs were synthesized through a hydrothermal method	Electrospun nanofibrous mats	MTT assay (L929 fibroblast cell line)	Male albino Wistar rats with full-thickness wounds	- Suitable physicochemical properties of the nanofibrous mats for cell adhesion and proliferation - Excellent antibacterial activity against *E. coli* and *S. aureus* - Low cytotoxicity to L929 fibroblasts - Enhanced wound healing by promoting wound contraction, enhancing collagen deposition, and accelerating re-epithelialization	[194]
CeO_2_ NPs/chitosan/cellulose acetate	Purchased from Sigma-Aldrich	Films prepared by solvent-casting method	–	–	- Improved mechanical and thermal properties - Suitable water vapor transmission rate - Antibacterial activity against *S. aureus* and *E. coli*	[183]
Gelatin/PCL/CeO_2_ NPs	Synthesized using cerium nitrate hexahydrate and hydrogen peroxide	Electrospun nanofibers	Alamar Blue assay (NIH-3T3 cells)	BALB/c mice with wounds infected by MRSA	- Enhanced proliferation of NIH-3T3 cells - Antibacterial activity against MRSA and *E.coli* - Accelerated wound closure - Inhibited bacterial growth *in vivo*	[195]
CeO_2_ NPs/chitosan/HEC/PEG	Purchased from Sigma-Aldrich	Films prepared by a solution casting method	–	–	- Dynamic mechanical properties improved due to the presence of CeO_2_ NPs - Good water vapor barrier properties - Desirable UV blocking properties - Antibacterial activity against *E. coli* and *S. aureus*	[196]
PVA/CeO_2_ NPs	Purchased from Sigma-Aldrich	Thermally crosslinked electrospun nanofibers	MTT and scratch assays (3T3 fibroblast cells)	–	- Suitable physicochemical and mechanical properties - Good biocompatibility, hemocompatibility and platelet adhesion, - Promoted cell migration in scratch assay	[197]
Gelatin/PCL/CeO_2_ NPs	Purchased from Sigma-Aldrich	Electrospun nanofibers	Annexin V/propidium iodide staining (human foreskin fibroblast HU2 cells)	–	- Slow release of CeO_2_ NPs - Down-regulated expression of SHV-betalactamase, KPC-carbapenemase, and metallo-β-lactamase IMP-1 genes involved in antibacterial resistance - Antibacterial activity against *Pseudomonas aeruginosa* - Cell proliferation increased with increasing CeO_2_ NPs concentration	[198]
GelMA/dopamine/AMP/CeO_2_ NPs	Purchased from US Research Nanomaterials	Sprayable hydrogel	CCK-8 assay and Live/Dead staining (HaCaT cells)	SD rats with full-thickness skin wounds infected with *S. aureus*	- Suitable physicochemical properties - Antibacterial activity against both gram-positive (*S. aureus* and *S. epidermidis*) and gram-negative (*P. aeruginosa* and *E. coli*) bacteria - High viability (>90%) of HaCaT cells cultured on the hydrogel - Excellent ROS-scavenging abilities - Enhanced wound healing rate of the infected wounds - Increased collagen I and collagen III deposition - Upregulated skin remodeling-associated gene expressions, including *SCD-1*, *LRIG-1*, and *PDGF-α* - Promoted remodeling of the healed skin	[143]
PCL/Gelatin/CeO_2_ NPs	Synthesized using cerium nitrate hexahydrate and hydrogen peroxide	Electrospun nanofibrous mesh	Alamar Blue assay (mouse embryo fibroblast 3T3-L1 cells)	–	- Enhanced proliferation of 3T3-L1 cells - SOD mimetic activity because of the incorporated CeO_2_ NPs - ROS scavenging activity	[199]
Gelatin/CeO_2_ NPs	Synthesized by thermal decomposition	Genipin-crosslinked Hydrogel	MTT and scratch assays (mouse embryonic fibroblast NIH-3T3 cells)	Female albino Wistar rats with excisional wounds	- Suitable physicochemical properties - No significant cell toxicity - Enhanced migration of NIH-3T3 fibroblast cells *in vitro* - Accelerated wound healing process - Higher tensile strength of the healed skin - Enhanced infiltration of leukocytes - Promoted angiogenesis - Increased collagen deposition	[200]
PCL/gelatin/CeO_2_ NPs	Purchased from Sigma-Aldrich	Electrospun films	MTT assay (L929 murine fibroblastic cell line)	Male Wistar rats with full-thickness excisional wounds	- Suitable physicochemical properties - High biocompatibility - Enhanced wound closure - Accelerated skin tissue regeneration	[201]
PCL/Pectin/CeO_2_ NPs	Synthesized using cerium nitrate and sodium hydroxide	Hydrogel prepared by casting	MTT assay (human fibroblast cells)	Male SD rats with 7-mm-thick wounds	- No cytotoxicity - Excellent antibacterial activity against *S. aureus* and *E. coli* - Antioxidant properties - Accelerated wound closure - Accelerated re-epithelialization - Increased collagen deposition	[202]
PU/Cellulose acetate/Zein/CeO_2_ NPs	Synthesized using polyvinylpyrrolidone and Ce(NO_3_)_3_·6H_2_O	Electrospun nanofibers	–	–	- Promoted platelets activation - Antibacterial activity against both Gram negative (*E. coli*, *Klebsiella pneumoniae*, and *Salmonella enterica*) and Gram positive (*S. aureus* and *Enterococcus faecalis*) bacteria	[203]
PU-cinnamon essential oil/PVA-gelatin-CeO_2_ NPs	Synthesized using Ce(NO_3_)_3_·6H_2_O and aqueous ammonia	Electrospun nanofibrous scaffolds prepared by a dual spinneret electrospinning technique	MTT assay and DAPI staining (adipose-derived mesenchymal stem cells)	–	- High porosity - High water uptake capacity - Suitable degradation rates - Appropriate mechanical properties for wound healing - Antibacterial activity against *S. aureus* and *E. coli* - No cytotoxicity	[204]
PCL/CeO_2_ NPs	Synthesized using Ce(NO_3_)_3_·6H_2_O and aqueous ammonia in the presence of gelatin	Electrospun nanofibers	DAPI staining, MTT assay, and LDH assay (hMSCs, HUVECs, and human neonatal foreskin fibroblast cells)	Male SD rats (scaffolds were subcutaneously implanted in the abdominal region for 4 weeks)	- High porosity - Sufficient mechanical strength and elasticity - Good hemocompatibility - Enhanced cell adhesion and subsequent proliferation due to the presence of CeO_2_ NPs - Increased the newly formed blood vessels in CAM assay - Enhanced angiogenesis *in vivo* - No systemic inflammation or mortality in SD rats - Upregulated expression of angiogenesis-related factors	[205]
Zein/CeO_2_ NPs/Ag–AgVO_3_	Synthesized using a hydrothermal method	Electrospun nanofibers	CCK-8 assay (L929 cells)	Male SD rats with full-thickness skin wounds	- Exhibited photocatalytic activity in the degradation of enrofloxacin - Exhibited antibacterial activity against both Gram-positive (*S. aureus* and *B. subtilis*) and Gram-negative (*E. coli* and *P. aeruginosa*) bacteria - Low cytotoxicity *in vitro* - Good *in vivo* biocompatibility	[206]
GelMA/xanthan gum/CMC	N-halamine-modified CeO_2_ NPs	3D-printed hydrogel dressings	CCK-8 assay (human pulmonary epithelial cells (A549 cells))	BALB/c mice with full-thickness wounds	- Excellent elasticity - High swelling ratio - Excellent antibacterial activity against *S. aureus* and *E. coli* - *In vitro* cytocompatibility - High hemocompatibility - Enhanced re-epithelialization - Accelerated the wound healing process	[191]

PVA: Poly(vinyl alcohol); CeO_2_ NPs: Cerium oxide nanoparticles; HDFs: Human dermal fibroblasts; MRSA: methicillin-resistant *Staphylococcus aureus*; GelMA: Gelatin methacryloyl; UV: Ultraviolet; MTT: 3-(4,5-Dimethylthiazol-2-yl)-2,5-diphenyltetrazolium bromide; SD rats: Sprague Dawley rats; PHBV: Poly(3-hydroxybutyrate-*co*-3-hydroxyvalerate); DAPI: 4′,6-diamidino-2-phenylindole; HOECs: Human oral epithelial cells; HMECs: human mammary epithelial cells; CAM: Chorioallantoic membrane; CBMA: 3-[[2-(Methacryloyloxy)ethyl] dimethylammonio]propionate; SBMA: [2-(methacryloloxy) ethyl]dimethyl-(3-sulfopropyl) ammonium hydroxide; HEMA: 2-Hydroxyethyl methacrylate; CCK-8: Cell counting kit-8; IL-6: Interleukin 6; PEO: Poly(ethylene oxide); HEC: Hydroxyethyl cellulose; PEG: Polyethylene glycol; PCL: Polycaprolactone; AMP: Antimicrobial peptide; ROS: Reactive oxygen species; SOD: Superoxide dismutase; PU: Polyurethane; hMSCs: Human mesenchymal stem cells; HUVECs: Human umbilical vein endothelial cells; AgVO_3_: Silver vanadium oxide; CMC: Carboxymethylcellulose sodium.

## 8 Bi- and multilayered scaffolds containing CeO_2_ NPs for wound healing

**Fig. 8:**
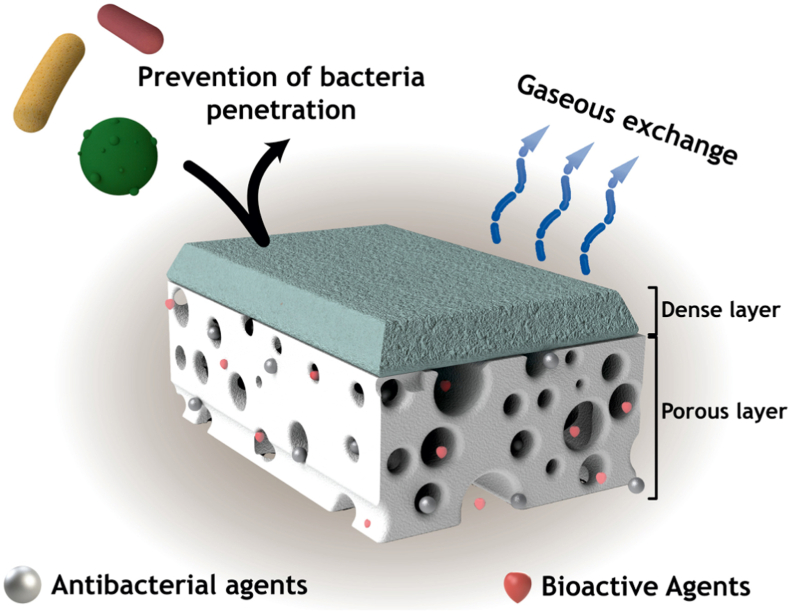
The main features of a suitable bi-layered construct for wound healing applications. Adapted and reprinted with permission from Elsevier [208].

Many injuries, as well as chronic wounds, usually involve both layers of the skin or even deeper tissues. As previously stated, the dermis layer of skin has a low cell density and a high amount of ECM produced by fibroblasts, the main cell population of this layer. Due to its structure, regeneration of the dermis is more complicated when compared to the epidermis. Therefore, novel approaches should be developed to improve regeneration quality based on the properties of each layer. For this purpose, bi-layered scaffolds have shown promising potential for wound treatment [22,207]. According to the literature, a bi-layered scaffold with a dense top layer and a porous underlying layer is more compatible with the treatment of full-thickness skin wounds. The top layer of such scaffolds is supposed to prevent bacterial infections and water loss at the wound site while also allowing for gaseous exchange [208,209]. The underlying porous layer should promote the proliferation and migration of fibroblasts, provide adequate space for ECM synthesis, support angiogenesis, have a high fluid absorption capacity, and facilitate tissue nutrition [210,211]. The incorporation of bioactive nanomaterials into such scaffolds could significantly improve their biological properties, such as angiogenic, anti-inflammatory, and anti-bacterial features [212,213]. Fig. 8 depicts a bi-layered construct with desirable properties for wound healing.

**Fig. 9:**
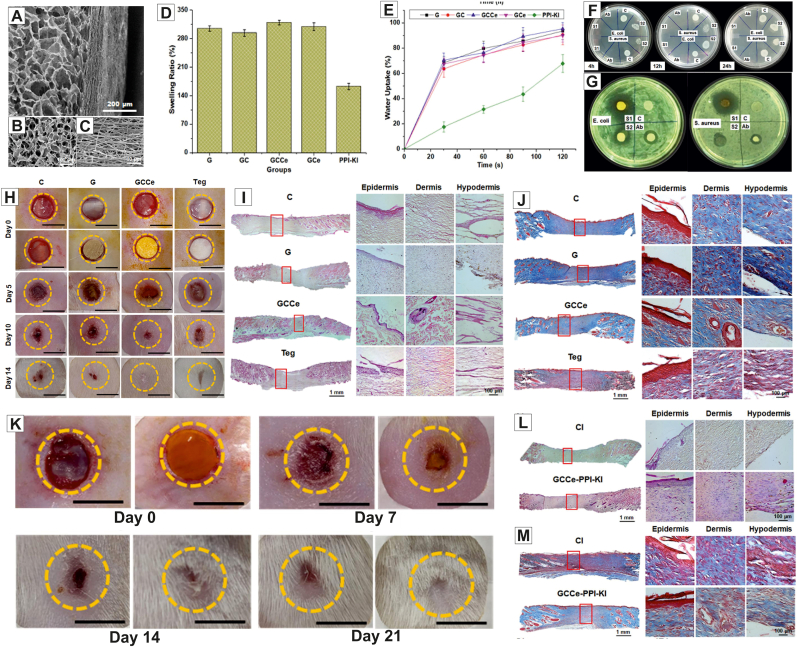
**A**) SEM image of the cross-section of the prepared gelatin cryogel-curcumin dextran nanoparticles-CeO_2_ NPs (GCCe)/polyvinyl alcohol-poly (vinyl pyrrolidone)-iodine-potassium iodide (PPI-KI) bilayer scaffold. **B**) SEM image of the surface morphology of the GCCe cryogel. **C**) SEM image of the surface morphology of PPI-KI nanofibers. **D**) Swelling ratio of the cryogel scaffolds (G: gelatin cryogel, GC: gelatin cryogel loaded with curcumin dextran nanoparticles, GCe: gelatin cryogel loaded with CeO_2_ NPs, GCCe: gelatin croygel loaded with both nanoparticles) and the PPI-KI nanofibrous membrane. **E**) Water uptake capacity of the cryogel scaffolds (G: gelatin cryogel, GC: gelatin cryogel loaded with curcumin dextran nanoparticles, GCe: gelatin cryogel loaded with CeO_2_ NPs, GCCe: gelatin cryogel loaded with both nanoparticles) and the PPI-KI nanofibrous membrane. **F**) Time-kill assay (C: Control, Ab: Antibiotic disc (1:1 gentamycin/penicillin), S1: PPI-KI nanofibrous membrane, and S2: PPI nanofibrous membrane). **G**) Disc diffusion test (C: Control, Ab: Antibiotic disc (1:1 gentamycin/penicillin), S1: PPI-KI nanofibrous membrane, and S2: PPI nanofibrous membrane). **H**) Macroscopic images of the full-thickness wounds, implantation of the scaffolds, and the wound closure rate during 14 days for each treatment (C: Control, G: gelatin cryogel, GCCe: gelatin cryogel loaded with both nanoparticles, Teg: Tegaderm wound dressing). **I**) Microscopic images of the regenerated skin tissues-H&E staining (C: Control, G: gelatin cryogel, GCCe: gelatin cryogel loaded with both nanoparticles, Teg: Tegaderm wound dressing). **J**) Masson's trichrome staining of the regenerated skin tissues (C: Control, G: gelatin cryogel, GCCe: gelatin cryogel loaded with both nanoparticles, Teg: Tegaderm wound dressing). **K**) The wound closure in the infected full-thickness wounds. **L**) Microscopic images of the regenerated skin tissues after the healing of infected wounds-H&E staining (CI: Control, GCCe-PPI-KI: The bilayer scaffold loaded with both nanoparticles). **M**) Masson's trichrome staining of the regenerated skin tissues after the healing of the infected wounds (CI: Control, GCCe-PPI-KI: The bilayer scaffold loaded with both nanoparticles). Adapted and reprinted with permission from Elsevier [214].

In a recent study, Andrabi et al. developed an antibacterial bilayer scaffold composed of gelatin cryogel loaded with curcumin dextran nanoparticles and CeO_2_ NPs as the underlying layer, and polyvinyl alcohol-poly(vinyl pyrrolidone)-iodine-potassium iodide (PPI-KI) electrospun nanofibers as the top layer for the treatment of infected and chronic wounds (Fig. 9). The prepared scaffold was hemocompatible and exhibited excellent water uptake capability and a high swelling ratio. The MTT assay results demonstrated the enhanced viability of NIH 3T3 cells. The scaffold also exhibited strong antioxidant activity. The antibacterial activity of the iodinated fibrous membranes against *E. coli* and *S. aureus* was confirmed using time-kill assays and disk diffusion tests, the results of which were consistent with the protective role of the outer layer. Further, the *in vivo* results demonstrated that gelatin cryogel loaded with curcumin dextran nanoparticles and CeO_2_ NPs (Group GCCe) significantly enhanced wound closure, accelerated re-epithelialization, promoted granulation tissue formation, reduced inflammatory response, increased collagen deposition, and induced angiogenesis during the healing process of full-thickness wounds. In another experiment, the bilayer scaffold was used to treat full-thickness wounds infected with *S. aureus* and *E. coli*. The bilayer scaffold accelerated the rate of healing in the infected wounds. Histological studies also revealed that the presence of the bilayer scaffold at the wound site enhanced the proliferation of fibroblasts, induced the formation of blood vessels, and increased collagen deposition [214].

The development of multilayer scaffolds with suitable properties supporting the regeneration of each layer can further promote wound healing and skin regeneration. Such scaffolds can be fabricated using different techniques such as 3D printing, lithography, molding, and electrospinning. Furthermore, appropriate drugs and factors can be loaded into each layer for local release, thus accelerating the healing rate even further [207,215]. PLA/PVA-CeO_2_ NPs/PLA trilayer nanofibrous membranes containing 1 or 2% w/w CeO_2_ NPs have been shown to be effective carrier systems for the treatment of chronic wounds due to the sustained release of CeO_2_ NPs [216].

## 9 Toxicity of CeO_2_ NPs

The use of nanoparticles in a wide range of fields has been growing in recent years; however, the toxicity of nanoparticles is currently a major concern. When it comes to biomedical applications, the assessment of nanoparticles toxicity is a critical factor that must be thoroughly addressed [217,218]. CeO_2_ NPs have been widely used in biomedical applications, including skin tissue engineering and wound healing studies. Despite promising results in a variety of studies, some studies have revealed the toxicity of these nanoparticles. Aalapati et al. demonstrated the accumulation of CeO_2_ NPs in pulmonary tissue and the pulmonary toxicity of these nanoparticles following nasal inhalation exposure in CD1 mice [219]. Wu et al. demonstrated the size-dependent pulmonary toxicity of CeO_2_ NPs after repeated intranasal instillation. The findings have revealed that CeO_2_ NPs with an average particle size of about 7 nm can cause more severe pulmonary damage than CeO_2_ NPs with an average particle size of about 25 nm [220]. Kumari et al. investigated the CeO_2_ NPs toxicity following repeated oral administration of different doses of the nanoparticles (30, 300, and 600 mg/kg/day) for 28 days in female Wistar rats. Following the administration of high doses of CeO_2_ NPs (300 and 600 mg/kg/day), they observed an increase in DNA damage in peripheral blood leukocytes and liver, changes in alkaline phosphatase and lactate dehydrogenase activity in serum, and reduced glutathione content in the kidneys, brain, and liver. Further histopathological studies revealed histological changes in spleen, brain, and liver [221]. According to the literature, the toxicity of CeO_2_ NPs depends on several factors, including concentration, nanoparticle size, exposure time, preparation method, exposure route, and cell/tissue type. Ji et al. synthesized a series of CeO_2_ nanorods and nanowires with precisely controlled lengths and aspect ratios to systematically determine the critical lengths and aspect ratios required to induce toxicity. *In vitro* toxicity investigations on human myeloid cell line (THP-1) demonstrated that CeO_2_ nanorods with lengths over 200 nm and aspect ratios above 22 caused progressive inflammatory effects and cytotoxicity. While lengths and aspect ratios play a role, the relatively low critical values identified were likely due to the small diameters of 6–10 nm, which allowed the nanorods to form stacking bundles through strong interactions, thus highlighting the importance of considering both length and diameter when assessing the cytotoxicity of high aspect ratio nanomaterials [222].

There are very limited published data on the adverse effects of CeO_2_ NPs on skin tissue and dermal cells. In this regard, Auffan et al. investigated the potential *in vitro* geno- and cytotoxicity of CeO_2_ NPs with an average size of about 7 nm on human dermal fibroblasts. The cytotoxicity of the nanoparticles was assessed using WST-1 cell viability assays. The results showed that 6 × 10^−2^ g/L of CeO_2_ NPs decreased the viability of human dermal fibroblasts. The genotoxicity of the nanoparticles was assessed by monitoring the formation of single-strand breaks in DNA and micronuclei induction. The findings showed that CeO_2_ NPs caused chromosome damage in a dose-dependent manner [223]. Cheng et al. evaluated the viability of HaCaT cells cultured in GelMA/dopamine-based hydrogels loaded with varying concentrations of CeO_2_ NPs (1, 10, 100, 1000 μg/mL, and 10 mg/mL) using the CCK-8 assay. The results indicated that hydrogels containing 100 μg/mL CeO_2_ NPs did not significantly affect the viability of the cells. However, hydrogels containing 1000 μg/mL and 10 mg/mL significantly reduced the viability of HaCaT cells, demonstrating the potential cytotoxicity of incorporated CeO_2_ NPs at high concentrations [143].

## 10 Conclusions

The management of skin wounds is currently a critical clinical issue, imposing high costs on patients and the medical system. Since wound healing is a complicated process affected by numerous factors, various approaches for promoting skin regeneration and accelerating wound healing have been developed. In this regard, a large number of studies have focused on nanobiomaterials as bioactive agents. CeO_2_ NPs have shown promising biological features, such as anti-oxidant, anti-inflammatory, antibacterial, and angiogenic properties, thus making these nanoparticles an excellent candidate for a wide range of biomedical applications, including skin tissue engineering and wound healing. These nanoparticles could be synthesized through various physical, chemical, and biological procedures. Today, the green synthesis of CeO_2_ NPs using plants, microbes, and food products has piqued the interest of researchers due to their abundance, high biocompatibility, cost-effectiveness, and environmental friendliness.

Along with the development of various methods for the green synthesis of CeO_2_ NPs, advances in skin tissue engineering have resulted in novel approaches that can promote wound healing in terms of rate and quality. CeO_2_ NPs can be incorporated into constructs fabricated by various techniques, such as electrospinning, solvent casting, freeze-drying, freeze-thawing, etc. The effectiveness of CeO_2_ NPs-containing scaffolds in promoting wound healing has also been demonstrated. Despite all such promising findings, the design and fabrication of constructs that can support the multifactorial nature of skin wound healing should be studied further. Bi- and multilayer scaffolds containing CeO_2_ NPs have demonstrated suitable properties for enhancing the regeneration of each layer of injured skin. However, the number of studies in this regard is limited, highlighting the need for further research.

Despite having excellent biological properties, information on the precise mechanism of action of CeO_2_ NPs is limited. There are concerns about their toxicity at high concentrations and long-term exposure. Furthermore, determining the optimal concentration of CeO_2_ NPs to promote the proliferation of native tissue cells and accelerate the healing process could be challenging. Incorporating these nanoparticles into scaffolds could prevent their aggregation and provide sustained release systems that can reduce the potential toxicity of CeO_2_ NPs; however, more research is needed to fully understand the underlying mechanisms and their effects on the wound healing process. To conclude, CeO_2_ NPs have the potential to play a critical role in meeting wound care challenges.

## Ethical approval

No experiments on human or animal subjects were performed in this study.

## CRediT authorship contribution statement

**Hamed Nosrati**: Conceptualization, Investigation, Writing – original draft, Writing – review & editing. **Morteza Heydari**: Investigation, Writing – original draft, Writing – review & editing. **Mohammad Khodaei**: Writing - Review & Editing.

## Funding

This research did not receive any specific grant from any funding agencies in the public, commercial, or not-for-profit sectors.

## Declaration of competing interest

The authors declare that they have no known competing financial interests or personal relationships that could have appeared to influence the work reported in this paper.

## Data Availability

No data was used for the research described in the article.
